# mRNA vaccines against SARS-CoV-2 induce comparably low long-term IgG Fc galactosylation and sialylation levels but increasing long-term IgG4 responses compared to an adenovirus-based vaccine

**DOI:** 10.3389/fimmu.2022.1020844

**Published:** 2023-01-12

**Authors:** Jana Sophia Buhre, Tamas Pongracz, Inga Künsting, Anne S. Lixenfeld, Wenjun Wang, Jan Nouta, Selina Lehrian, Franziska Schmelter, Hanna B. Lunding, Lara Dühring, Carsten Kern, Janina Petry, Emily L. Martin, Bandik Föh, Moritz Steinhaus, Vera von Kopylow, Christian Sina, Tobias Graf, Johann Rahmöller, Manfred Wuhrer, Marc Ehlers

**Affiliations:** ^1^ Laboratories of Immunology and Antibody Glycan Analysis, Institute of Nutritional Medicine, University of Lübeck and University Medical Center Schleswig-Holstein, Lübeck, Germany; ^2^ Center for Proteomics and Metabolomics, Leiden University Medical Center, Leiden, Netherlands; ^3^ Institute of Nutritional Medicine, University of Lübeck and University Medical Center Schleswig-Holstein, Lübeck, Germany; ^4^ Department of Anesthesiology and Intensive Care, University of Lübeck and University Medical Center Schleswig-Holstein, Lübeck, Germany; ^5^ Medical Department 2, University Heart Center of Schleswig-Holstein, Lübeck, Germany; ^6^ Airway Research Center North (ARCN), University of Lübeck, German Center for Lung Research (DZL), Lübeck, Germany

**Keywords:** vaccination, antibody, SARS-CoV-2, COVID-19, IgG, IgG subclass, IgG glycosylation, IgA

## Abstract

**Background:**

The new types of mRNA-containing lipid nanoparticle vaccines BNT162b2 and mRNA-1273 and the adenovirus-based vaccine AZD1222 were developed against SARS-CoV-2 and code for its spike (S) protein. Several studies have investigated short-term antibody (Ab) responses after vaccination.

**Objective:**

However, the impact of these new vaccine formats with unclear effects on the long-term Ab response – including isotype, subclass, and their type of Fc glycosylation – is less explored.

**Methods:**

Here, we analyzed anti-S Ab responses in blood serum and the saliva of SARS-CoV-2 naïve and non-hospitalized pre-infected subjects upon two vaccinations with different mRNA- and adenovirus-based vaccine combinations up to day 270.

**Results:**

We show that the initially high mRNA vaccine-induced blood and salivary anti-S IgG levels, particularly IgG1, markedly decrease over time and approach the lower levels induced with the adenovirus-based vaccine. All three vaccines induced, contrary to the short-term anti-S IgG1 response with high sialylation and galactosylation levels, a long-term anti-S IgG1 response that was characterized by low sialylation and galactosylation with the latter being even below the corresponding total IgG1 galactosylation level. Instead, the mRNA, but not the adenovirus-based vaccines induced long-term IgG4 responses – the IgG subclass with inhibitory effector functions. Furthermore, salivary anti-S IgA levels were lower and decreased faster in naïve as compared to pre-infected vaccinees. Predictively, age correlated with lower long-term anti-S IgG titers for the mRNA vaccines. Furthermore, higher total IgG1 galactosylation, sialylation, and bisection levels correlated with higher long-term anti-S IgG1 sialylation, galactosylation, and bisection levels, respectively, for all vaccine combinations.

**Conclusion:**

In summary, the study suggests a comparable “adjuvant” potential of the newly developed vaccines on the anti-S IgG Fc glycosylation, as reflected in relatively low long-term anti-S IgG1 galactosylation levels generated by the long-lived plasma cell pool, whose induction might be driven by a recently described T_H1_-driven B cell response for all three vaccines. Instead, repeated immunization of naïve individuals with the mRNA vaccines increased the proportion of the IgG4 subclass over time which might influence the long-term Ab effector functions. Taken together, these data shed light on these novel vaccine formats and might have potential implications for their long-term efficacy.

## Introduction

The rapid spread of severe acute respiratory syndrome coronavirus type 2 (SARS-CoV-2), the cause of Coronavirus Disease 2019 (COVID-19), has led to a global health threat (1). The virus employs a transmembrane spike (S) protein that interacts through its receptor-binding domain (RBD) with the host membrane-bound angiotensin-converting enzyme 2 (ACE2) to enter cells of the respiratory tract (2). A range of different intramuscularly administered vaccines inducing an immune response against the S-protein have been developed, led by novel mRNA-containing lipid nanoparticle (LNP) vaccines such as the BNT162b2 and the mRNA-1273 vaccines from BioNTech/Pfizer (3) and Moderna (4), respectively, as well as replication-deficient adenovirus-based vaccines such as the ChAdOx1 nCoV-19 (AZD1222) vaccine from AstraZeneca (5).

Neutralizing anti-S IgG and IgA antibodies (Abs) inhibiting the interaction of the viral S protein with ACE2 have been identified both in blood and in the respiratory tract after SARS-CoV-2 infection (6–9). Persisting neutralizing Abs in the respiratory tract likely constitute the first line of defense that protect from a subsequent SARS-CoV-2 re-infection and spreading (8, 10).

Although both mRNA vaccines induce strong initial neutralizing anti-S IgG and IgA Ab responses in the blood, all three vaccines seem to be only moderately or temporarily protective against SARS-CoV-2 infection and spreading (3, 7, 9–15). Moreover, better vaccine-induced protection from infection and spreading has been described for previously (pre)-infected vaccinees (with SARS-CoV-2 history) as compared to naïve vaccinees (without SARS-CoV-2 history) (16, 17). However, long-term anti-S IgG and IgA levels in the blood and the respiratory tract have hardly been investigated in naïve and pre-infected subjects upon vaccination against SARS-CoV-2.

Nevertheless, all three vaccines seem to induce high protection from severe disease conditions in the next weeks after a second immunization (5, 18, 19), assuming a robust long-term systemic T and B cell response – also against non-RBD parts of the virus and virus escape variants (15). However, the influence of the different new vaccine formats with unclear co-stimulatory/”adjuvant” effects on the long-term B cell and Ab Fc response remains unknown.

IgG Fc-mediated effector functions are influenced by the induced IgG subclass and the IgG Fc *N*-glycosylation pattern. Human IgG1 and IgG3 subclasses have been described to convey the highest potential to activate immune cells *via* classical activating Fcγ receptors (FcγRs) and the classical complement pathway *via* C1q (20–24). These IgG subclasses can form hexamers, thereby facilitating the interaction with the six-arm C1q molecule (21, 25–29). IgG2 hardly interacts with classical FcyRs and C1q and its effector function-inducing capacity needs further investigation (20, 22, 23). In contrast, IgG4 shows higher affinity to the classical IgG inhibitory receptor FcyRIIB than to classical activating FcyRs (20, 22, 23). Furthermore, IgG4 cannot activate C1q but instead is able to disturb the hexamer formation of the C1q-activating IgG subclasses (21). Furthermore, IgG4 can generate Fab arm-exchange, meaning that heavy chains with different specificities can dimerize resulting in bispecific Abs, which reduces their ability to form immune-complexes (30). Thus, IgG1 and IgG3 are the IgG subclasses with the highest potential to activate the immune system, whereas IgG4 has less activating potential and can even inhibit the effector functions of IgG1 and IgG3.

Both SARS-CoV-2 infection and vaccination initially induce the IgG1 and IgG3 subclasses against the S protein (5, 31–35).

Another factor known to influence IgG Fc-mediated effector functions is the type of IgG Fc *N-*glycosylation. The highly conserved glycosylation site at Asn297 in the Fc moiety of IgG carries a complex type *N*-glycan characterized by a core structure, that can be further modified with a core fucose, a bisecting *N-*acetylglucosamine (GlcNAc) as well as one or two galactose residues, each of which can further be capped by a sialic acid (36–38) (**Table S2**).

IgG Abs lacking fucose are known to have an increased affinity to activating FcyRIIIa and are linked to enhanced tumor-fighting potential as well as protection against HIV and malaria infection (38–43).

Agalactosylated (G0) IgG Abs have been linked to severe conditions in inflammatory (auto-) immune diseases, whereas IgG sialylation has been associated with a decreased affinity of IgG to classical activating FcyRs and lower or anti-inflammatory effects (21, 24, 36, 37, 44–52). The functional analysis of differently glycosylated IgG Abs is complex because single terminal glycan residues may in addition interact with glycan binding receptors, such as galectins, siglecs, and C-type lectin receptors (37, 44, 53–55). *In vivo*, immune inhibitory functions have been described for sialylated as well as terminally galactosylated antigen-specific and total IgG Abs (36, 44–48, 52, 54, 56). IgG Fc bisection is often increased in inflammatory autoimmune diseases (57). However, the biological significance of IgG bisection is less clear and remains to be investigated.

In the context of SARS-CoV-2 infection, transient afucosylated and more persistent agalactosylated anti-S IgG (1) Abs have been linked to pro-inflammatory disease conditions in COVID-19 patients without (non-ICU), but in particular in those with the need of intensive care unit (ICU) admission (32–35, 58–61). Noteworthy, severe COVID-19 conditions have also been linked to the appearance of broad autoreactive IgG Abs (62). It remains unclear whether a combination of inflammatory Fc glycosylation patterns and broad auto-reactivity of the induced IgG Abs contributes to the severe inflammatory complications of COVID-19. However, it has rather been investigated whether the inflammatory IgG glycosylation patterns contribute to the inflammatory complications than to the elimination of the virus. Afucosylated as well as agalactosylated anti-S IgG Abs may also strengthen the anti-viral response (63).

A recent mouse vaccination study with different adjuvants showed that the early, likely extrafollicularly induced IgG Abs were characterized by high Fc galactosylation and sialylation levels (64). Over time, the potential of each adjuvant/co-stimulus to induce an IFNγ- and IL-17-producing T follicular helper (T_FH1_ and T_FH17_) cell-dependent germinal center (GC) B cell and Ab response became visible that correlated with lower long-term IgG Fc galactosylation and sialylation levels (64). A recent vaccination study against simian immunodeficiency virus in rhesus macaques with two different adjuvant formats found comparable results. In that study, the adjuvant, which correlated with better protection, also correlated with lower induced IgG galactosylation and sialylation levels (65).

The new mRNA vaccines against SARS-CoV-2 generate an initial anti-S and -RBD IgG (1) Ab response with transient afucosylation, but high galactosylation and sialylation levels (32, 34, 35).

However, little is known about repeated immunizations and the potential of the different new vaccine formats with unclear “adjuvant” effects on the long-term IgG subclass and IgG Fc glycosylation response.

Here, we present a comprehensive analysis of serum-derived and salivary anti-S1 IgG (subclass) and IgA Ab responses as well as anti-S serum IgG1 Fc *N*-glycosylation patterns of SARS-CoV-2 naïve and pre-infected individuals vaccinated with different vaccine combinations over time to compare and characterize the long-term Ab responses up to day 270 post-immunization.

## Materials and methods

### Study cohort

SARS-CoV-2 naïve (with no known SARS-CoV-2 history) and non-hospitalized pre-infected (with past SARS-CoV-2 infection history) subjects were recruited at the University of Lübeck and the University Medical Center Schleswig-Holstein (Lübeck, Germany) since December 2020. The regimens for six vaccination cohorts varied as follows: (i) 48 naïve individuals received two doses of the BioNTech/Pfizer vaccine BNT162b2 (each 30 µg) (3); (ii) 25 naïve individuals received two doses of the Moderna vaccine mRNA-1273 (each 100 µg) (4); (iii) 14 naïve individuals received two doses of the adenovirus-based vaccine ChAdOx1 nCoV-19 (AZD1222) from AstraZeneca (each 5x10^10^ virus particle with not less than 2.5x10^8^ infectious units) (5); (iv) 12 naïve individuals received one dose of AZD1222 and subsequently one dose of BNT162b2; (v) 44 naïve individuals received one dose of AZD1222 and subsequently one dose of mRNA-1273; and (vi) 14 non-hospitalized pre-infected individuals received one or two doses of BNT162b2 (**Table 1** and **Table S1**).

**Table 1:** Vaccination study groups.

Group number	Naïve or pre-infected	First and second vaccine		Abbreviation and colour code of the vaccination study groups used in the figures
		First vaccine	Second vaccine	
1	naïve	BNT162b2	BNT162b2	**B**+**B**
2	naïve	mRNA-1273	mRNA-1273	**M**+**M**
3	naïve	AZD1222	AZD1222	**A**+**A**
4	naïve	AZD1222	BNT162b2	**A**+**B**
5	naïve	AZD1222	mRNA-1273	**A**+**M**
6	pre-infected	BNT162b2	(BNT162b2)*	**B**+(B)*

*some of the pre-infected individuals only got one vaccination up to day 270 after the first vaccination.

The mRNA and adenovirus vaccinees received their second dose between day 21 and 45 or between day 70 and 84 (except for five vaccinees with AZD1222 that received their second dose between day 35 and 61), respectively, and were sampled (blood serum and/or saliva) once or multiple times up to 270 days after the first immunization.

In addition to pre-vaccination samples of the pre-infected subjects, 2 further non-hospitalized pre-infected non-vaccinated individuals as positive controls, and 8 non-vaccinated naïve subjects as negative controls were recruited.

No selection criteria were used and participants as well as repeated sampling were selected at random. However, the preferred vaccination strategies at the University Medical Center Schleswig-Holstein and the University of Lübeck were vaccination with two doses of BNT162b2 or the first vaccination with AZD1222 followed by a booster injection of mRNA-1273, respectively, explaining the comparably high numbers of individuals/samples in these two groups.

The identification of pre-infected individuals was limited by their low incidence in the catchment area when the project was started in December 2020 and the number of vaccinated pre-infected individuals was even lower because of unclear recommendations regarding vaccination after infection.

All recruited pre-infected individuals have had a mild pre-infection meaning that they neither had to go to the hospital (non-hospitalized) nor showed signs of shortness of breath or abnormal chest imaging during infection. Furthermore, most recruited pre-infected individuals were vaccinated with BNT162b2 and not with mRNA-1273 or AZD1222. So, we included only BNT162b2-vaccinated pre-infected in this study. Most of these pre-infected individuals were tested positive for SARS-CoV-2 in a narrow time window between 200 and 150 days before the first vaccination (only three individuals were infected earlier). This is why we decided not to investigate the influence of this period on the vaccine-induced Ab response.

To verify and recognize pre-infected individuals, previous positive SARS-CoV-2 PCR results were considered, together with anti-viral nucleocapsid protein (anti-NCP) and anti-S1 serum IgG responses (**Figure S1F**).

Blood samples and saliva were collected after obtaining written informed consent according to the Declaration of Helsinki in accordance with the local ethics board-approved protocol 20-123 (Ethics Committee of the University of Lübeck, Germany).

### Blood serum and saliva antibody detection

Blood samples were collected as described earlier (31). Salivary samples were collected with the Saliva Collection system-ORACOL Plus S14 (Malvern Medical Developments, United Kingdom) and frozen before usage.

Enzyme-linked Immunosorbent Assays (ELISA) were used to detect always anti-S1 (the extracellular part of S containing the RBD) Abs. The EUROIMMUN SARS-CoV-2 S1 IgG (EUROIMMUN, Lübeck, Germany; #EI 2606-9601-2 G), the EUROIMMUN SARS-CoV-2 S1 IgA (#EI 2606-9601-2 A), and the EUROIMMUN SARS-CoV-2-NCP IgG (#EI 2606-9601-2 G) ELISA were performed according to manufacturer’s instructions; serum dilution: 1/101. A ratio to reference value was calculated by dividing the sample OD (450 nm) value by the OD (450 nm) value of a reference sample provided by the manufacturer.

Alternatively, 96-well ELISA plates were coated with 4 µg/mL of SARS-CoV-2-S1 antigen (ACROBiosystems, Newark, DE 19711, USA; #S1N-C52H3) to identify anti-S1 serum IgG and IgG1-4 (Hansestadt Lübeck (HL)-1 ELISA), or anti-S1 salivary IgG and IgA (HL-2 ELISA) levels as recently described (31), or J-chain-coupled salivary Abs (HL-2 ELISA). These HL ELISA protocols were established in-house. Briefly (HL-1 and HL-2 ELISA), the plates were washed with 0.05% Tween 20 in PBS to remove unbound antigens. In case of anti-S1 salivary IgG and IgA detection additional blocking (HL-2 ELISA) was performed with 0.05% Tween 20, and 3% BSA in PBS. Subsequently, serum (diluted 1/1000 for IgG and IgG1, 1/100 for IgG2-4, and in addition 1/10 for IgG1 (**Figure S2B**) detection) or saliva (diluted 1/10 for IgG and IgA detection) in 0.05% Tween 20, 3% BSA in PBS were added. Bound Abs were detected with horseradish peroxidase (HRP)-coupled polyclonal goat anti-human IgG Fc (#A80-104P) or IgA (#A80-102P)-specific Abs purchased from Bethyl Laboratories (Montgomery, TX, USA) or monoclonal anti-human IgG1 (clone HP-6001), IgG2 (clone HP-6014), IgG3 (clone HP-6050), or IgG4 (clone HP-6025)-specific Abs purchased from Southern Biotech (Birmingham, AL, USA) or anti-J-chain Ab (clone F-12) obtained from Santa Cruz Biotechnology (Dallas, TX, USA; #sc-133177) in 0.05% Tween 20, 3% BSA in PBS. After incubation with the 3,3′,5,5′-tetramethylbenzidine (TMB) substrate (BD Biosciences, San Diego, CA, USA), the optical density (OD) was measured at 450 nm. Secondary Ab specificity was verified recently (31). OD (450 nm) values are shown or alternatively, a ratio to reference value was calculated by dividing the sample OD (450 nm) value through the OD (450 nm) value of an internal reference sample of an individual with a historic non-hospitalized SARS-CoV-2 infection.

### IgG Fc glycosylation analysis

Total IgG Abs were affinity-captured from sera using Protein G Sepharose 4 Fast Flow beads (GE Healthcare, Uppsala, Sweden) in a 96-well filter plate (Millipore Multiscreen, Amsterdam, Netherlands), as described (60, 66). Eluates from total IgG affinity-purification were dried by vacuum centrifugation and subjected to tryptic cleavage followed by liquid chromatography (LC)-mass spectrometry (MS) analysis according to established procedures (60). Using this method, IgG1 glycoforms were assigned based on accurate mass and specific migration position in LC, excluding the possible glycopeptide-level interference of IgG3 with IgG2 and IgG4 (66).

### LC-MS data processing

Raw LC-MS spectra were converted to mzXML files. LaCyTools, an in-house developed software was used for the alignment and targeted extraction of raw data (67). Alignment was performed based on the average retention time of at least three highly abundant glycoforms. The analyte list for targeted extraction of the 2^+^ and 3^+^ charge states was based on manual annotation as well as on literature reports (60, 64).

The inclusion of an analyte for the final data analysis was based on quality criteria including signal-to-noise (higher than 9), isotopic pattern quality (less than 25% deviation from the theoretical isotopic pattern), and mass error (within ±20 parts per million range) leading to a final analyte list (**Table S2**). The relative intensity of each glycan form in the final analyte list was calculated by normalizing it to the sum of their total glycoform areas. Normalized intensities were used to calculate fucosylation, bisection, galactosylation, and sialylation (**Tables S2**, **S3**). Serum samples with low anti-S Ab levels shortly after the first vaccination did not always result in sufficient signal strengths and hence were excluded from the analysis.

Anti-S IgG1 Fc N-glycosylation patterns from unvaccinated, hospitalized non-ICU and ICU SARS-CoV-2 patients were used for comparison. Therefore, anti-S IgG1 Fc N-glycopeptide raw data of our previous study (60) were used to calculate the glycosylation traits based on the analyte list described above (**Tables S2**, **S3**).

### Statistical analysis

Statistical analyses were performed using GraphPad Prism v6.0 and v9.0 (GraphPad, La Jolla, CA), and MatLab (The MathWorks Inc., Massachusetts, NE). The smoothed mean curves shown in scatter plots were created by polynomial regression. Confidence bands were plotted by using a confidence level of 95%. Data in bar graphs were presented as mean values ± SD. Differences between two groups were assessed with the Mann-Whitney U test. Differences between more than two groups were assessed with the Kruskal-Wallis test. Pearson correlation was done to measure the strength of the linear relationship between two variables. *p*-values < 0.05 were considered significant as follows: *, **, ***, ****: *p*-value < 0.05, 0.01, 0.001, and 0.0001 respectively. Principal component analyses (PCA) were performed in GraphPad Prism v9.0, while the partial least square-discriminant analyses (PLS-DA) were performed in PLS-Toolbox (Eigenvector Research Inc., Wenatchee, WA) in MatLab. For cross-validation, venetian blinds were used and the area under the receiver operating characteristic curve (AUROC) was calculated.

## Results

### Six study groups

Naïve and non-hospitalized SARS-CoV-2 pre-infected individuals were recruited in Lübeck, Germany, that received one of the six mRNA and adenovirus-based SARS-CoV-2 vaccine combinations shown in **Table 1**, and sampled (blood serum and/or saliva) once or multiple times up to 270 days after the first immunization.

### Anti-S1 serum and salivary IgG Ab responses

**Figure 1:**
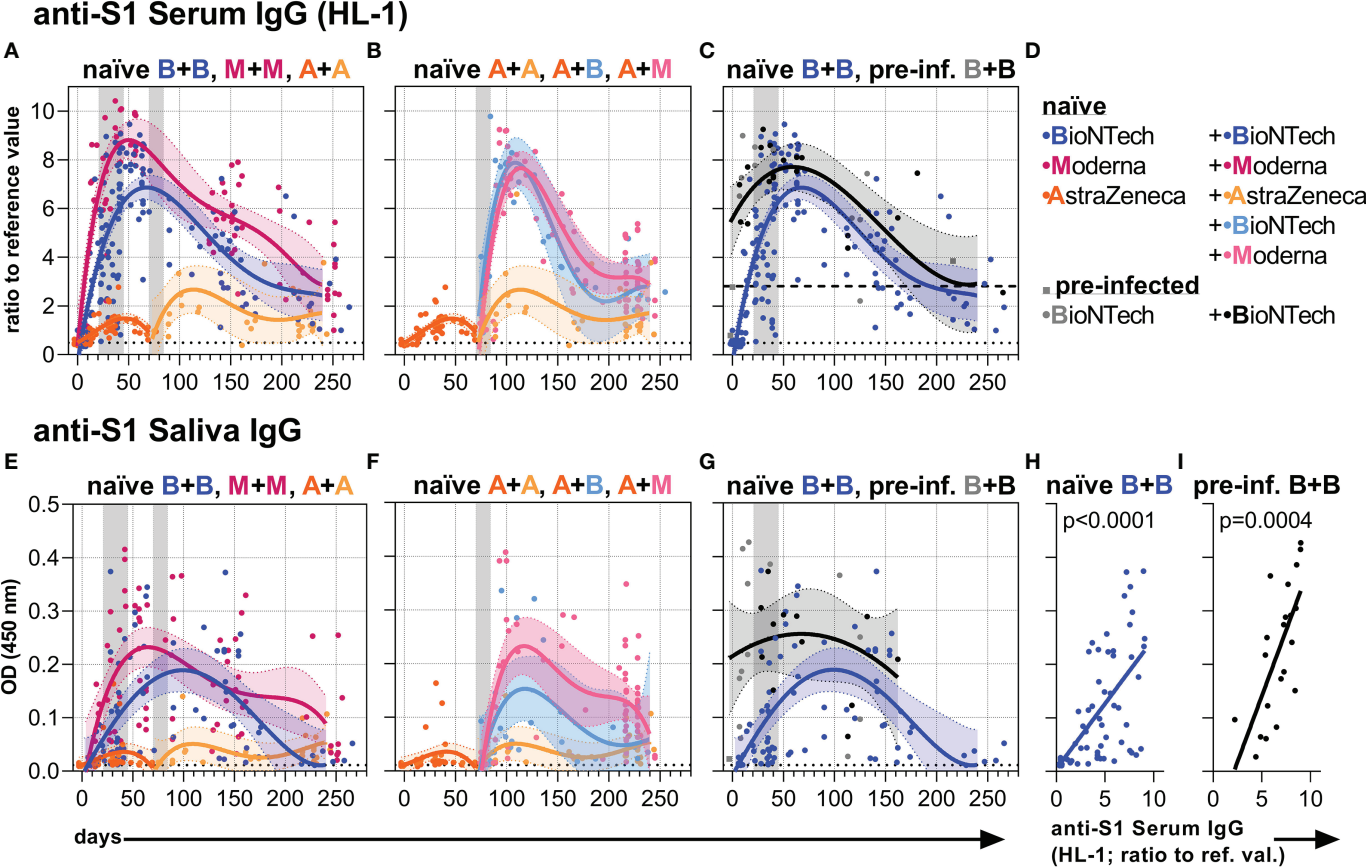
Anti-S1 serum and salivary IgG levels. **(A-C)** Anti-S1 serum IgG (HL-1 ELISA) levels (ratios to reference value) of the indicated six vaccination groups. **(D)** Color legend of the six study groups. **(E–G)** Anti-S1 salivary IgG levels (OD 450 nm values) of the indicated six groups. Gray bars: time windows of the second shot after the first shot with an mRNA (between day 21 and 45) or adenovirus-based (between day 70 and 84) vaccine. Dashed (— **(C)**) and dotted (…) lines indicate the corresponding average anti-S1 IgG levels of pre-infected individuals without/before vaccination or non-vaccinated healthy (negative) controls, respectively. **(H, I)** Pearson correlations between anti-S1 serum IgG (HL-1) levels and anti-S1 salivary IgG levels (y-axis as in **(E)**) of all paired samples from once and twice BNT162b2-vaccinated naïve and pre-infected individuals. *p*-values of the indicated correlations are shown.

First, we analyzed anti-S1 serum IgG Ab levels by commercially available (EUROIMMUN) and in-house developed (HL) ELISA methods (**Figures 1A–D**, **S1**). Early anti-S1 serum IgG levels of naïve and pre-infected vaccinees were similar to those described earlier (9, 31, 34, 68). All three vaccines induced higher anti-S1 serum IgG levels after the second as compared to the first vaccination indicating a re-activation of memory B cells (**Figures 1A**, **S1**). Two vaccinations with an mRNA vaccine induced higher anti-S1 serum IgG levels than two vaccinations with the adenovirus-based vaccine AZD1222 (**Figures 1A**, **S1**). Among the mRNA vaccines, mRNA-1273 appeared to induce higher anti-S1 IgG titers than BNT162b2 (**Figures 1A**, **S1**). Individuals who received the first immunization with the adenovirus-based vaccine and the second with either one or the other mRNA vaccine reached IgG levels comparable to the levels induced with two mRNA vaccinations (**Figures 1B**, **S1**). High anti-S1 serum IgG levels were observed early on after the first vaccination with BNT162b2 in pre-infected individuals (**Figures 1C**, **S1**).

Subsequently, however, the high mRNA-induced anti-S1 serum IgG levels of naïve and pre-infected individuals waned and approached over time the long-term levels observed following two adenovirus-based vaccinations (**Figures 1A–C**, **S1**). Furthermore, anti-S1 IgG time courses were similar for and highly correlated between serum and saliva (**Figures 1E–I**, **S1**) assuming passive transfer of IgG between blood and lumen/mucosa of the respiratory tract (8, 69).

### Anti-S1 serum IgG subclass responses

**Figure 2:**
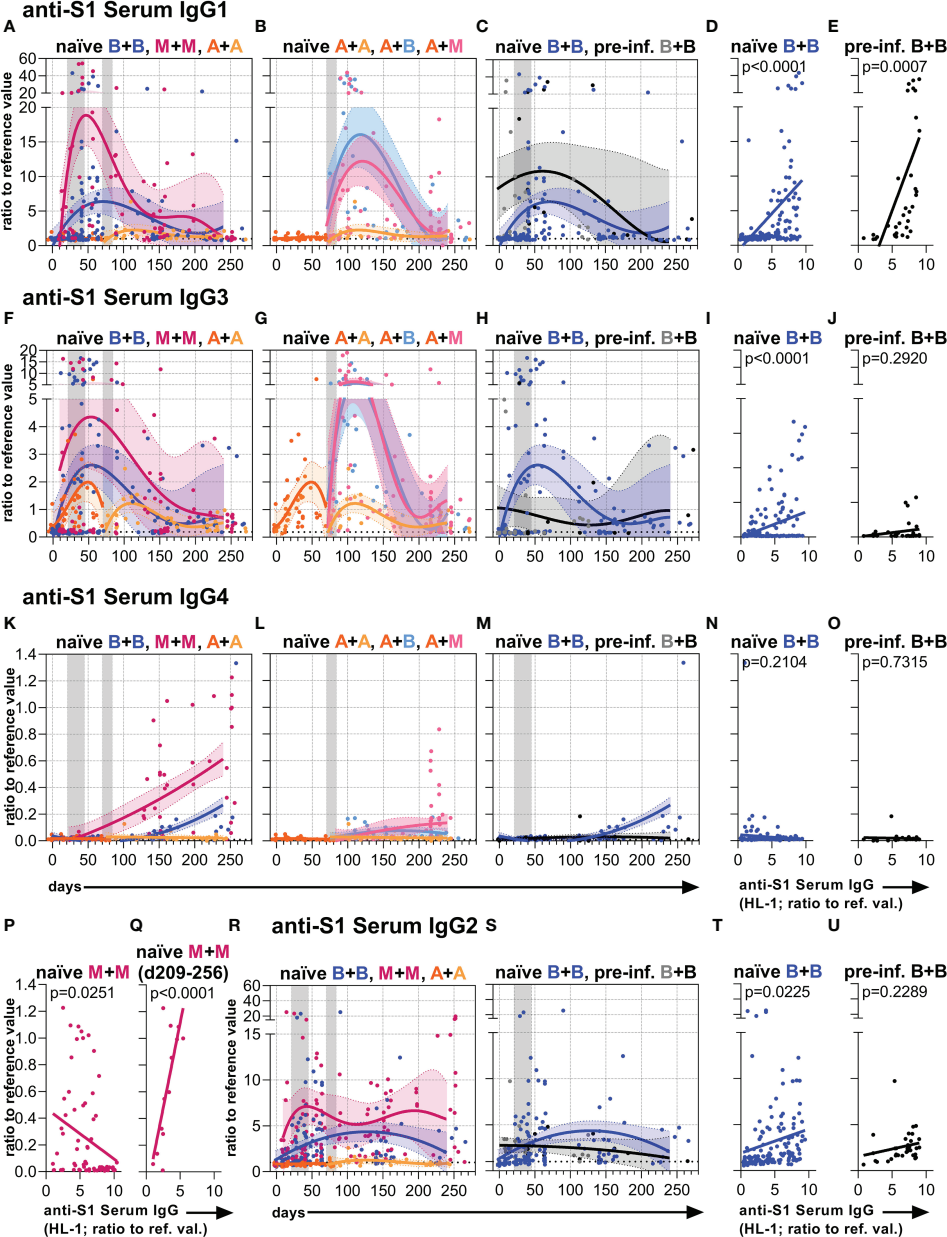
Anti-S1 serum IgG1-4 subclass levels. Anti-S1 serum **(A–C)** IgG1, **(F–H)** IgG3, **(K–M)** IgG4, and **(R, S)** IgG2 levels (ratios to reference values) of the indicated six vaccination groups. The A+B and A+M groups were not analyzed for IgG2. The used color codes are identical to **Figure 1D**. Gray bars: time windows of the second shot after the first shot with an mRNA (between day 21 and 45) or adenovirus-based (between day 70 and 84) vaccine. Dotted lines indicate the corresponding average anti-S1 IgG1, IgG3, IgG4, or IgG2 levels of non-vaccinated healthy (negative) controls. **(D, E, I, J, N–Q, T, U)** Pearson correlations between anti-S1 serum IgG levels (HL-1) and anti-S1 serum **(D, E)** IgG1, **(I, J)** IgG3, **(N–Q)** IgG4, or **(T, U)** IgG2 levels (y-axis as in (**A, F, K** or **R**, respectively)) of all paired samples from one and two-times BNT162b2-vaccinated **(D, I, N, T)** naïve and **(E, J, O, U)** pre-infected individuals, and **(P, Q)** two-times mRNA-1273-vaccinated naïve individuals. The IgG to IgG4 correlation in **(Q)** was only done with paired long-term samples of two times mRNA-1273-vaccinated naïve individuals collected between day (d) 209 and 256 upon the first immunization. *p*-values of the indicated correlations are shown.

Next, we analyzed anti-S1 serum IgG subclass abundances over time (**Figures 2**, **S2**, **S3**). Our observations confirmed recent findings describing that the mRNA vaccines initially induce anti-S1 IgG1 followed by IgG3 and IgG2 and hardly any IgG4 responses (31, 32, 34), whereas vaccination with AZD1222 mainly results in anti-S IgG1 and IgG3, but hardly any IgG2 and IgG4 (5) in the first weeks after immunization of naïve individuals (**Figures 2**, **S2**, **S3**).

Furthermore, we observed more intense differences in early anti-S1 IgG1 than in early IgG3 levels between the naïve mRNA and AZD1222 groups, with the high initial mRNA-induced anti-S1 IgG response dominated by IgG1 (**Figures 2A, F**, **S2**). Over time, anti-S1 IgG1 and IgG3 levels became comparable between the different naïve vaccination groups (**Figures 2A, B, F, G**, **S2**).

Interestingly, the two times mRNA-1273 vaccination group and to a lesser extent also the two times BNT162b2 vaccination group generated long-term IgG4 responses (**Figures 2K**, **S3**). This long-term IgG4 response also developed in vaccinees receiving a combination of AZD1222 and mRNA-1273 as well as AZD1222 and BNT162b2 but to a lesser extent (**Figures 2L**, **S3**). Notably, the two times AZD1222 vaccination group did not show this long-term IgG4 response (**Figures 2K, L**, **S3**). The initial IgG2 response after mRNA vaccination kept higher over time as compared to the adenovirus-based vaccination in naïve individuals (**Figures 2R, S**, **S3**).

In pre-infected individuals mostly an increase of IgG1 levels was observed after vaccination with BNT162b2. Although this group showed comparable long-term IgG subclass levels when compared to naïve individuals immunized with BNT162b2 (**Figures 2C, H, M, S**, **S2**, **S3**), their IgG4 response seemed not to be or barely induced (**Figures 2M, O** **)**.

The anti-S1 serum IgG1-3 levels of naïve and the IgG1 levels of pre-infected vaccinees positively correlated with their total anti-S1 serum IgG levels, whereas the IgG2-4 levels of pre-infected vaccinees did not show such a significant positive correlation (**Figures 2D, E, I, J, T, U**, **S2**, **S3**). In contrast, the anti-S1 serum IgG4 levels of naïve individuals vaccinated with mRNA vaccines showed a significant or in tendency negative correlation with their total anti-S1 serum IgG levels (**Figures 2N–P**, **S3**). However, anti-S1 serum IgG4 levels of naïve individuals vaccinated twice with mRNA-1273 significantly correlated with their total anti-S1 serum IgG levels, when only long-term samples (between day 209-256 upon the first immunization) were considered (**Figures 2Q**, **S3**).

Thus, two immunizations or at least a second immunization with an mRNA vaccine generated detectable long-term IgG4 responses in naïve individuals. Notably, the mRNA-1273 vaccine showed a higher potential to generate such a late IgG4 response than the BNT162b2 vaccine.

### Anti-S1 serum and salivary IgA Ab responses

**Figure 3:**
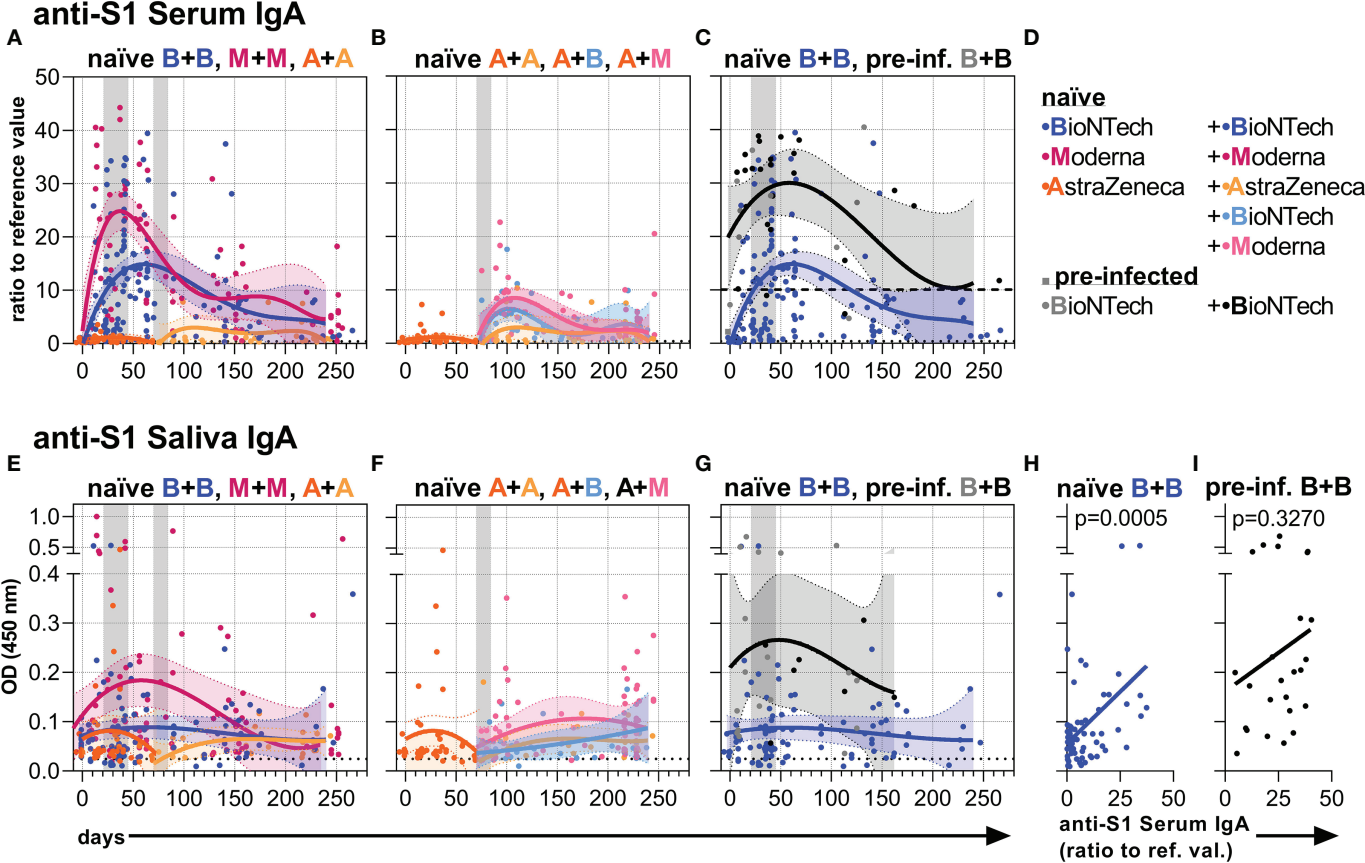
Anti-S1 serum and salivary IgA levels. **(A-C)** Anti-S1 serum IgA levels (ratios to reference value) of the indicated six vaccination groups. **(D)** Color legend of the six study groups. **(E-G)** Anti-S1 salivary IgA levels (OD 450 nm values) of the indicated six groups. Gray bars: time windows of the second shot after the first shot with an mRNA (between day 21 and 45) or adenovirus-based (between day 70 and 84) vaccine. Dashed and dotted lines indicate the corresponding anti-S1 IgA average levels of pre-infected individuals without/before vaccination or non-vaccinated healthy (negative) controls, respectively. **(H, I)** Pearson correlations between anti-S1 serum IgA and salivary IgA levels (y-axis as in **(E)**) of all paired samples from once and twice BNT162b2-vaccinated naïve and pre-infected individuals. *p*-values of the indicated correlations are shown.

In na**ï**ve individuals, two vaccinations with mRNA-1273 induced higher anti-S1 IgA levels in the serum and saliva than BNT162b2, and both mRNA vaccines induced higher levels than two doses with AZD1222 (**Figures 3A, E, D**, **S4**). In contrast to the anti-S1 IgG levels, the anti-S1 IgA levels in serum and saliva of naïve individuals seemed to be boosted less and decreased faster (**Figures 3A, E**, **S4**). Further, the mRNA vaccines induced only a reduced anti-S1 IgA response as compared to the anti-S1 IgG response when the individuals were first vaccinated with AZD1222 (**Figures 3B, F**, **S4**). Over time, the mRNA vaccine-induced IgA levels gradually approached the rather low IgA levels resulting from two AZD1222 vaccinations (**Figures 3A, E**, **S4**).

In contrast, pre-infected individuals vaccinated with BNT162b2 reached and maintained higher anti-S1 IgA levels both in serum and saliva over time compared to naïve individuals vaccinated with BNT162b2 (**Figures 3C, G**, **S4**). Anti-S1 saliva IgA levels correlated in pre-infected as well as naïve vaccinees with anti-S1 saliva J-chain levels (**Figure S4**) suggesting that the anti-S1 salivary IgA is mostly dimeric J-chain-coupled secretory (s)IgA in all groups. While anti-S1 salivary IgA levels correlated with anti-S1 serum IgA levels in naïve vaccinees, they did not display such a significant correlation in pre-infected ones (**Figures 3H, I**, **S4**). The findings suggest a proper, but more decoupled re-activation of local respiratory and systemic S1-reactive IgA^+^ B cells in pre-infected vaccinees.

### Anti-S serum IgG1 Fc *N-*glycosylation

**Figure 4:**
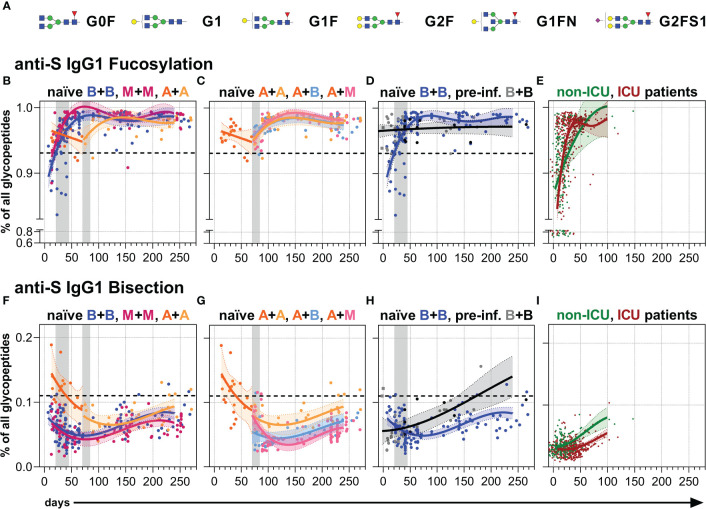
Anti-S serum IgG1 fucosylation and bisection. **(A)** The six major IgG Fc *N*-glycans attached to Asn 297 of IgG1 with an average relative abundance of more than 3% (**Table S2**): Galactose: G, yellow circle; sialic acid: S, purple diamond; fucose: F, red triangle; mannose: green circle; N-acetylglucosamine: GlcNAc and bisecting GlcNAc, N, blue square. **(B–D)** Anti-S serum IgG1 Fc *N*-fucosylation and **(F–H)** anti-S serum IgG1 Fc *N*-bisection of the indicated six vaccination groups. The used color codes are identical to **Figure 1D**. Gray bars: time windows of the second shot after the first shot with an mRNA (between day 21 and 45) or adenovirus-based (between day 70 and 84) vaccine. Dashed lines indicate the average level of total IgG1 Fc fucosylation or bisection, respectively (**Figure S6**). **(E)** Anti-S serum IgG1 Fc *N*-fucosylation and **(I)** anti-S serum IgG1 Fc *N*-bisection of unvaccinated, hospitalized non-ICU and ICU SARS-CoV-2 patients from our previous study (60) for comparison.

**Figure 5:**
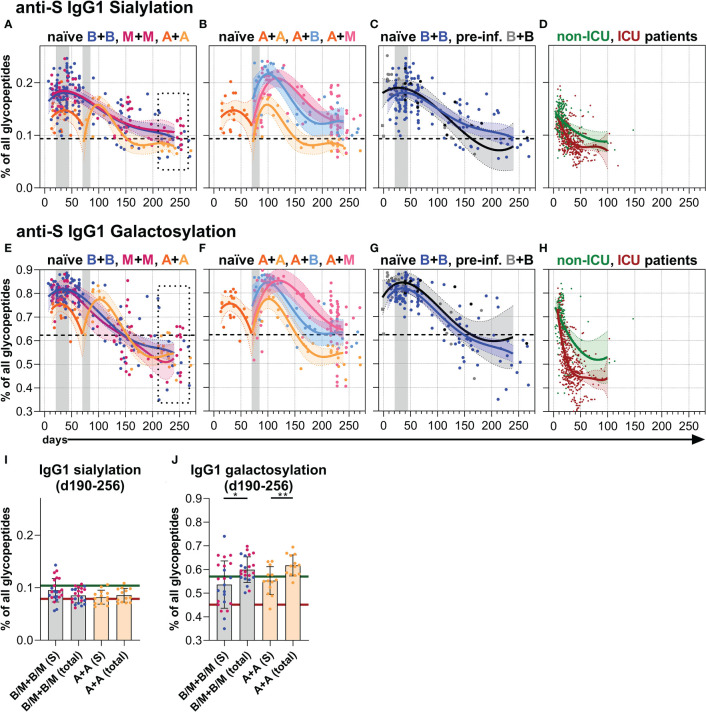
Anti-S serum IgG1 sialylation and galactosylation. **(A–C)** Anti-S serum IgG1 Fc *N*-sialylation and **(E–G)** anti-S serum IgG1 Fc *N*-galactosylation of the indicated six vaccination groups. The used color codes are identical to **Figure 1D**. Gray bars: time windows of the second shot after the first shot with an mRNA (between day 21 and 45) or adenovirus-based (between day 70 and 84) vaccine. Dashed lines indicate the average level of total IgG1 Fc sialylation and galactosylation, respectively (**Figure S6**). **(D)** Anti-S serum IgG1 Fc *N*-sialylation and **(H)** anti-S serum IgG1 Fc *N*-galactosylation of unvaccinated, hospitalized non-ICU and ICU SARS-CoV-2 patients from our previous study (60) for comparison. **(I, J)** Comparison of anti-S and total (**Figure S6**) IgG1 **(I) **sialylation and **(J)** galactosylation levels of samples from mRNA (B/M+B/M; B: blue; M: pink) or adenovirus (A+A) vaccinated individuals collected late between day (d) 190 and 256 upon first immunization. The green and red lines indicate the mean of the anti-S IgG1 sialylation and galactosylation levels from the hospitalized non-ICU and ICU patients, respectively, upon day 50 post-infection **(D, H)**.

Finally, we analyzed the Fc *N*-glycosylation patterns of anti-S and total serum IgG1 over time up to day 270 by LC-MS (**Figures 4**, **5**, **S5**, **S6** and **Tables S2**, **S3**). The analysis resulted in the identification of 12 IgG1 Fc glycopeptide species (the six major glycan species are schematically shown in **Figure 4A**), from which glycosylation traits of fucosylation, bisection, sialylation, and galactosylation were calculated (**Tables S2**, **S3**). The development of the anti-S IgG1 glycosylation from the vacinees were compared to the anti-S IgG1 glycosylation from unvaccinated, hospitalized non-ICU and ICU SARS-CoV-2 patients investigated in the context of our previous study (60) (**Figures 4E, I**, **5D, H**).

#### Fucosylation

In line with recent findings, the first immunization with BNT162b2 induced an initial anti-S IgG1 response with a temporarily afucosylated glycosylation pattern, with fucosylation levels as low as 80% (34) (**Figures 4B**, **S5**). The fucosylation levels steadily increased in the next few weeks to about 98%, surpassing the corresponding total IgG1 fucosylation levels (92-94%), and stagnated over the study period (**Figures 4B**, **S5**, **S6**). A comparable longitudinal fucosylation pattern was observed upon one and two mRNA-1273 immunizations (**Figures 4B**, **S5**, **S6**). On the other hand, one immunization with AZD1222 seemed to induce a less pronounced early afucosylated anti-S IgG1 response, only down to 95% fucosylation, that also increased upon a booster immunization, irrespective of the vaccine type, to about 98% (**Figures 4B, C**, **S5**, **S6**).

In contrast, BNT162b2-vaccinated non-hospitalized pre-infected individuals maintained stable anti-S IgG1 fucosylation levels over time (96-97%), which was lower than the long-term anti-S IgG1 fucosylation level of naïve vaccinees and might have had developed already before vaccination as hypothesized previously (34) (**Figures 4D**, **S5**, **S6**). In comparison, the early, very low anti-S IgG1 fucosylation levels (down to 70% fucosylation) of the unvaccinated hospitalized patients vastly increased and reached fucosylation levels higher than 95% already 50 days post-infection (60) (**Figure 4E**).

#### Bisection

Following booster immunization with an mRNA vaccine, anti-S IgG1 bisection levels had fallen, then slightly increased, but remained below their total counterpart in naïve individuals throughout the study period (34) (**Figures 4F**, **S5**, **S6**).

One dose of AZD1222 induced higher early anti-S IgG1 bisection levels than one dose of an mRNA vaccine (**Figures 4F**, **S5**) but after a second immunization irrespective of the vaccine type the levels became comparable to the levels upon two mRNA doses (**Figures 4G**, **S5**, **S6**). However, the two times AZD1222 vaccination group showed a slightly higher long-term anti-S IgG1 bisection level than the other vaccine combinations throughout the study period (**Figures 4F, G**, **S5**). Upon day 100, the anti-S IgG1 bisection levels of pre-infected vaccinees surpassed those of naïve individuals upon two BNT162b2 vaccinations (**Figures 4H**, **S5**, **S6**). The unvaccinated, hospitalized non-ICU and ICU patients showed very low early anti-S IgG1 bisection levels that increased over time (60) (**Figure 4I**).

#### Galactosylation and sialylation

Both one and two immunizations of naïve individuals with an mRNA vaccine led to initial anti-S IgG1 sialylation as well as galactosylation levels higher than their corresponding total IgG1 sialylation and galactosylation levels, a finding consistent with our recent report (34) (**Figures 5A, E**, **S5**, **S6**). However, these anti-S IgG1 sialylation and galactosylation levels decreased with the passage of time and the anti-S1 galactosylation levels even fell below their corresponding total IgG1 levels, but still remained above the very low anti-S1 IgG1 galactosylation levels of the unvaccinated, hospitalized ICU patients that prevailed upon day 50 post-infection (60) (**Figures 5A, E, D, H, I, J**, **S5**, **S6**).

Although the initial anti-S IgG1 galactosylation and sialylation levels were lower after one AZD1222 dose compared to one mRNA vaccine dose, the AZD1222-induced levels upon a second dose with AZD1222, BNT162b2, or mRNA-1273 became comparable to the mRNA-induced ones over time up to day 270 (**Figure 5**, **S5**, **S6**). BNT162b2-vaccinated, pre-infected individuals showed a comparable anti-S IgG1 sialylation and a slightly higher anti-S IgG1 galactosylation course to naïve individuals vaccinated with BNT162b2 (**Figures 5C, G**, **S5**, **S6**).

### Potential predictive parameters and long-term differences in the antibody response upon vaccination

**Figure 6:**
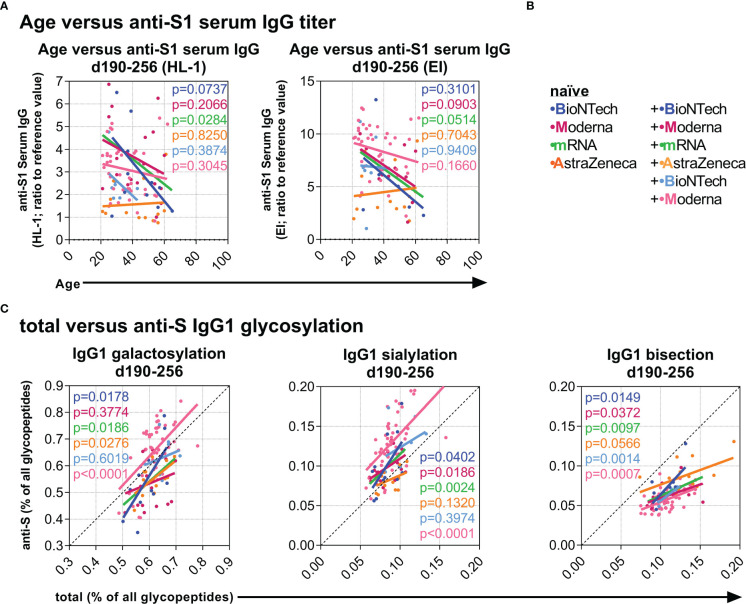
Investigation of potential predictive parameters. **(A)** Negative correlations between age and late anti-S1 IgG titers (samples collected between day (d) 190 and 256 upon first vaccination) (Left: HL-1 ELISA data; Right: EUROIMMUN (EI) ELISA data) of the indicated vaccination groups. **(B)** Color legend of the six naïve groups; the mRNA group includes both naïve B+B- and M+M-vaccinated individuals. **(C)** Positive correlations between total and anti-S IgG1 galactosylation, sialylation, and bisection levels from samples collected late between day (d) 190 and 256.

Next, we explored associations between the data at baseline and at later time points to identify potential predictive correlations. Interestingly, age negatively correlated with the long-term anti-S1 serum IgG levels between day 190 and 256 in naïve individuals after two mRNA vaccinations but not after two AZD1222 vaccinations (**Figures 6A, B**). Furthermore, total IgG1 galactosylation, sialylation, and bisection levels positively correlated significantly or in tendency with the long-term anti-S IgG1 galactosylation, sialylation, and bisection levels, respectively, between day 190 and 256 of individuals from all groups (**Figure 6C**), suggesting an influence of the total IgG galactosylation, sialylation, and bisection levels on the induced anti-S T and B cell responses in all vaccination groups.

**Figure 7:**
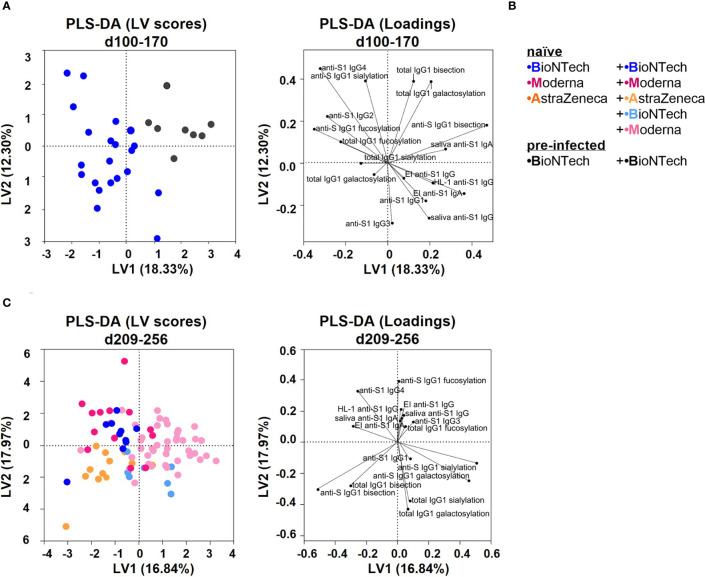
Partial least squares-discriminant analysis (PLS-DA) of the study groups **(A)** PLS-DA of data collected from the naïve and pre-infected B+**(B)** vaccination cohorts between day (d) 100 and 170 upon first vaccination. Latent variable (LV) scores and loadings are shown. The area under the receiver operating characteristic curve (AUROC) for cross-validation is 0.8810 for both groups. **(B)** Color legend of the six vaccination groups. **(C)** PLS-DA of data collected from all naïve vaccination cohorts between day (d) 209 and 256 upon first vaccination. The AUROC values for cross-validation are B+B: 0.8304, M+M: 0.8449, A+A: 0.7416, A+B: 0.8773, and A+M: 0.9094.

Finally, we verified long-term outcomes between the vaccination groups by performing principal component analyses (PCA) and partial least square-discriminant analyses (PLS-DA) (**Figures 7**, **S7**). Conversely to PCA, PLS-DA considers the initial separation in vaccination groups. Both analyses resulted in comparable parameter-dependent separations, albeit with slightly stronger separations with the PLS-DA analysis (**Figures 7**, **S7**).

Naïve and pre-infected vaccinees that received two BNT162b2 immunizations were separated between day 100 and 170 as follows: pre-infected vaccines showed higher anti-S1 serum and saliva IgA as well as anti-S IgG1 bisection and galactosylation levels, whereas naïve vaccines showed higher anti-S1 IgG4 and anti-S IgG1 fucosylation levels (**Figures 7A, B**, **S7**).

Naïve individuals that received two AZD1222 immunizations were separated between day 209 and 256 by higher anti-S IgG1 bisection levels and lower anti-S1 IgG4 titers from two times mRNA vaccinated individuals (**Figures 7C**, **S7**). AZD1222 vaccinees that received an mRNA booster were separated between day 209 and 256 by higher anti-S1 Ab titers and anti-S IgG1 galactosylation and sialylation levels, likely because of later boosting (**Figures 7C**, **S7**).

## Discussion

Our study shows that the mRNA-containing LNP vaccines BNT162b2 and mRNA-1273 induce high anti-S1 IgG and IgA levels in the blood as well as in the saliva, but these Ig levels steadily decrease over time and approach levels that are comparable to the long-term levels induced by two immunizations with the adenovirus-based vaccine AZD1222. In the long run, such pronounced anti-S1 IgG and (s)IgA reductions in the saliva likely reflect the declining protection against infection and from spreading in the respiratory tract of naïve individuals (16, 17). On the other hand, the observed stronger anti-S1 (s)IgA response in the saliva of previously infected vaccinees – likely generated by re-activation of infection-induced local (s)IgA^+^ memory B cells – might explain their recently described higher protection from infection and spreading (16, 17, 70).

Neutralizing mucosal Abs play a crucial role in preventing infections of the respiratory tract. Therefore, optimized vaccination strategies against pathogens of the respiratory tract should enhance local antigen-specific long-lived PC and memory B cell responses for generating an improved, long-lasting Ab frontline defense response in the mucosa of the respiratory tract (71–73).

Both mRNA- and adenovirus-based vaccines generate comparable long-term anti-S1 IgG1 and IgG3 levels up to day 270; whereas the IgG2 levels remained higher after mRNA vaccination. Very interestingly, two mRNA immunizations as well as one AZD1222 immunization with an mRNA booster, in particular with the mRNA-1273 vaccine, induced long-term anti-S1 IgG4 responses – the IgG subclass with inhibitory effector functions – in naïve subjects. In contrast, we could not observe such an increase upon two immunizations with the AZD1222 vaccine in naïve individuals up to day 270, suggesting that only mRNA vaccines generate detectable long-term IgG4 responses at least until day 270.

Supporting evidence stems from another study, where comparable results were observed, additionally with a further increase of IgG4 after the third vaccination with BNT162b2 (74). Similarly to our results, no IgG4 response was detected after two AZD1222 vaccinations. The authors observed a slight IgG4 response after immunization with AZD1222 and boosting with BNT162b2, while the effects of the mRNA-1273 vaccine were not analyzed.

In a setting of HIV vaccination, a study compared repeated immunizations with two related HIV vaccine formats. The authors described that the protection of one vaccine composition correlated with the induction of IgG1 and IgG3 Abs, whereas the other vaccine composition hardly showed any protection, which correlated with the generation of IgG4 upon repeated immunizations instead (75). Interestingly, repeated immunizations by allergen-specific immunotherapy also induce IgG4 responses over time (50). Thus, the vaccine composition might influence the IgG4-inducing capacity upon repeated immunization.

The mRNA-1273 vaccine (100 µg) contained higher amounts of mRNA than the BNT162b2 vaccine (30 µg) that will influence the amount and probably also the duration of S protein expression. One or both circumstances might contribute to the higher induction of anti-S1 IgG4 by the mRNA-1273 vaccine. Furthermore, the induced IgG4 class switching might occur directly from IgM or *via* other IgG subclass intermediates, as the *Igg4* gene locus is the most downstream of the IgG subclass gene loci.

Notably, in pre-infected individuals no IgG4 responses were observed upon one or two doses of BNT162b2. However, since the case number and observation period for pre-infected individuals were limited, further studies involving more long-term samples are needed to verify this observation. In the future, the potential non-neutralizing effects of IgG4 on the elimination of SARS-CoV-2 require further investigation.

Two mRNA vaccinations and also an AZD1222 vaccination with an mRNA booster induce massive, but temporary anti-S1 IgG(1) responses, presumably generated by short-term PCs. Accordingly, IgG Abs generated by long-term PC responses become noticeable only after several months, when the short-lived PC responses had already faded. In contrast, the adenovirus-based vaccine induced a weaker short-term Ab response, and Abs generated by long-term PC subsets likely become dominant more rapidly.

Early and late IgG responses may convey functionally divergent roles. Recent studies suggest that highly galactosylated and sialylated short-term IgG responses might facilitate antigen-delivery to GC reactions in a sialylation-dependent manner for aiding affinity maturation and therewith the induction of IgG Abs with high neutralizing potential (76, 77). In contrast, long-term IgG Ab responses with lower galactosylation and sialylation levels might thereby induce a stronger immune cell activation, also, after subsequent infection with emerging SARS-CoV-2 variants, when neutralizing capacities of the existing Abs are diminished due to their potentially reduced RBD-specificities (15).

Although the fighting potential of differently galactosylated and sialylated IgG Abs against pathogens has to be further investigated, it is important to comprehend how vaccine compositions influence IgG Fc galactosylation and sialylation levels in both short- and long-term PC responses (78).

A recent mouse immunization study has shown that the (inflammatory) potential of an adjuvant (co-stimulus) reflects the qualitative potential of a vaccine composition to determine the IgG Fc galactosylation and sialylation levels during the GC response and thereby the GC-derived long-term IgG response (64). In summary, the results suggested distinct adjuvant-specific (inflammatory) potentials of different adjuvants to induce GC-driven antigen-specific IgG Abs with low galactosylation and sialylation levels: CFA (complete Freund’s adjuvant; water-in-oil adjuvant+*M.tb.*) > IFA, Montanide (both water-in-oil adjuvants) > Alum (aluminum hydroxide) > AddaVax (similar to MF59; squalene-based), Toll-like-receptor ligands (64).

Furthermore, the adjuvant Alum induced better protection from subsequent SIV infection than MF59, correlating with Alum-induced anti-gp120 IgG Abs with lower galactosylation and sialylation levels than MF59 (65).

Mechanistically, the induction of antigen-specific IgG Abs with low galactosylation and sialylation levels in the GC (e.g. with IFA) was linked to the induction of IL-6-dependent IFNγ-producing T follicular helper T_FH1_ cells. CFA-induced very low IgG galactosylation and sialylation levels have further been linked to the additional induction of (inflammatory) IL-17-producing T_FH17_ cells (64).

Strong inflammatory immune responses induced in ICU-admitted SARS-CoV-2-infected patients were characterized by high IL-6 and IL-17 levels (79), CCR6^+^ circulating (c)T_FH17_ cells in the blood (80, 81), and anti-S IgG1 Abs with very low galactosylation levels that prevailed upon day 50 post-infection (60) (**Figure 5H**). In contrast, non-hospitalized SARS-CoV-2 patients were characterized by CXCR3^+^ cT_FH1_ cells (82), and relatively higher anti-S IgG1 galactosylation and sialylation levels (34).

The mRNA and adenovirus-based vaccines induced comparable, relatively low long-term anti-S IgG1 galactosylation levels both in naïve vaccinees and vaccinees with past infection. These anti-S IgG1 galactosylation levels were lower than their corresponding total IgG1 galactosylation levels, but not as low as the presumably pro-inflammatory anti-S IgG1 galactosylation levels of ICU patients that were prevailing upon day 50 post-infection (60).

These findings suggest that the adenovirus-based and the two mRNA-containing LNP vaccine formats have a comparable, strong potential to influence the quality of the long-term anti-S IgG (1) response, as reflected by the low long-term anti-S IgG1 Fc galactosylation levels.

The stimulatory “adjuvant” potential” of the adenovirus-based vaccine might be induced by the activation of pattern-recognition receptors (PRRs) on immune cells, due to its adenovirus-inherent activation nature. Although the mRNAs in the mRNA vaccines have been modified to reduce their interaction with PRRs, residual activation of PRRs cannot be excluded and potentially co-stimulate immune cells. In addition, it has been described that LNPs can also have a co-stimulatory adjuvant effect (83, 84).

Recent mouse studies have shown that an mRNA-containing LNP vaccine is inducing rather a T_FH1_- than an IL-4-producing T_FH2_-driven GC response, whereas an RBD protein-AddaVax vaccination rather induced a T_FH2_-driven GC response (85). Furthermore, both mRNA vaccines have induced GC B cells and T_FH1_ > T_FH2_, but hardly T_FH17_ cells in human lymph nodes (86) as well as cT_FH1_ cells in the blood (82). For the adenovirus-based vaccine, a strong T_H1_ response has been described (87).

Altogether, these human data suggest, comparable to the murine data described above (64), that the induction of (c)T_FH17_ > (c)T_FH1_ > (c)T_FH2_ cells might correlate with lower long-term antigen-specific IgG Fc galactosylation and sialylation levels upon vaccination. Accordingly, the moderate/relatively low long-term anti-S IgG galactosylation levels in the three vaccination groups seem to correlate with the recently described (c)T_FH1_-driven B cell response for all three vaccines.

Predictively, age correlated with lower long-term IgG Ab titers for the mRNA vaccines. Furthermore, total IgG1 galactosylation, sialylation, and bisection levels correlated with higher long-term anti-S IgG1 sialylation, galactosylation, and bisection levels, respectively, for all vaccine combinations suggesting that total IgG Fc glycosylation patterns might influence the glycosylation patterns of antigen-specific immune responses upon vaccination.

In summary, the data indicate that the high initial mRNA vaccine-induced anti-S1 IgG(1) and IgA responses decrease over time and approach levels induced with the adenovirus-based vaccine up to day 270. Higher and more stable anti-S1 (s)IgA levels in the saliva of pre-infected vaccinees might explain their higher protection from infection and spread of SARS-CoV-2.

Intriguingly, the mRNA vaccines, and in particular the mRNA-1273 vaccine, induced increasing long-term anti-S1 serum IgG4 levels in naïve individuals with hitherto unclear influences on the fight against the pathogen. Naïve individuals vaccinated with the adenovirus-based vaccine did not show such long-term anti-S1 IgG4 response at least after two vaccinations until day 270.

Instead, both the mRNA-containing LNP and adenovirus-based vaccines induced comparable anti-S IgG1 glycosylation responses over time up to day 270 as reflected in relatively low anti-S IgG(1) galactosylation levels. This low galactosylation level might reflect the stimulatory “adjuvant” potential of the new vaccine formats and a previously described, primarily T_H1_-driven B cell response, which overall may contribute to the described efficient protection from severe infections. Understanding the long-term adjuvant effects of mRNA and adenovirus-based vaccinations against SARS-CoV-2 will have potential implications on future vaccine designs.

## Data availability statement

The original contributions presented in the study are included in the article/**Supplementary Material**. Further inquiries can be directed to the corresponding authors.

## Ethics statement

The studies involving human participants were reviewed and approved by Ethics Committee of the University of Lübeck, Germany. The patients/participants provided their written informed consent to participate in this study.

## Author contributions

Organization of blood and saliva sampling: JB, IK, AL, BF, EM, SL, MS, VK, JR, CS, TG, and ME. Serum and saliva ELISA analysis: JB, IK, AL, EM, SL, HL, CK, LD, JP, and JR. IgG glycosylation analysis: TP, WW, JN, and MW. Statistical analysis: JB, FS, CS, JR, TP, and ME. Supervision: MW and ME. Writing - original draft: JB, TP, MW, and ME. Initial submission: JB. All authors contributed to the article and approved the submitted version.
