# Supplementary material for: mRNA vaccines against SARS-CoV-2 induce comparably low long-term IgG Fc galactosylation and sialylation levels but increasing long-term IgG4 responses compared to an adenovirus-based vaccine
Source: Front Immunol. 2023 Jan 12;13:1020844. doi: 10.3389/fimmu.2022.1020844 (PMC9877300; doi:10.3389/fimmu.2022.1020844)
Supplement: Supplementary file 1 [file Presentation_1.pdf]

Supplementary Material

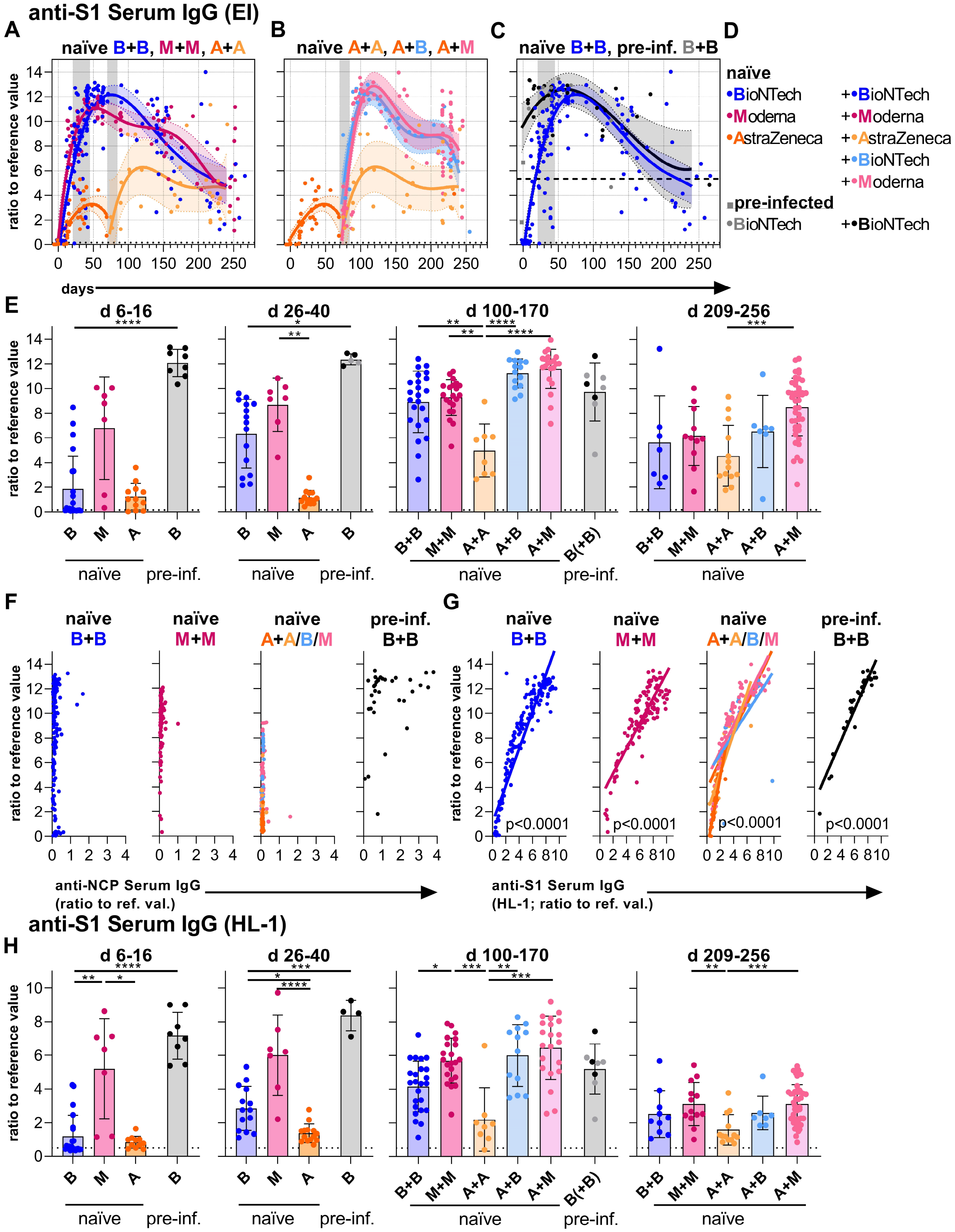

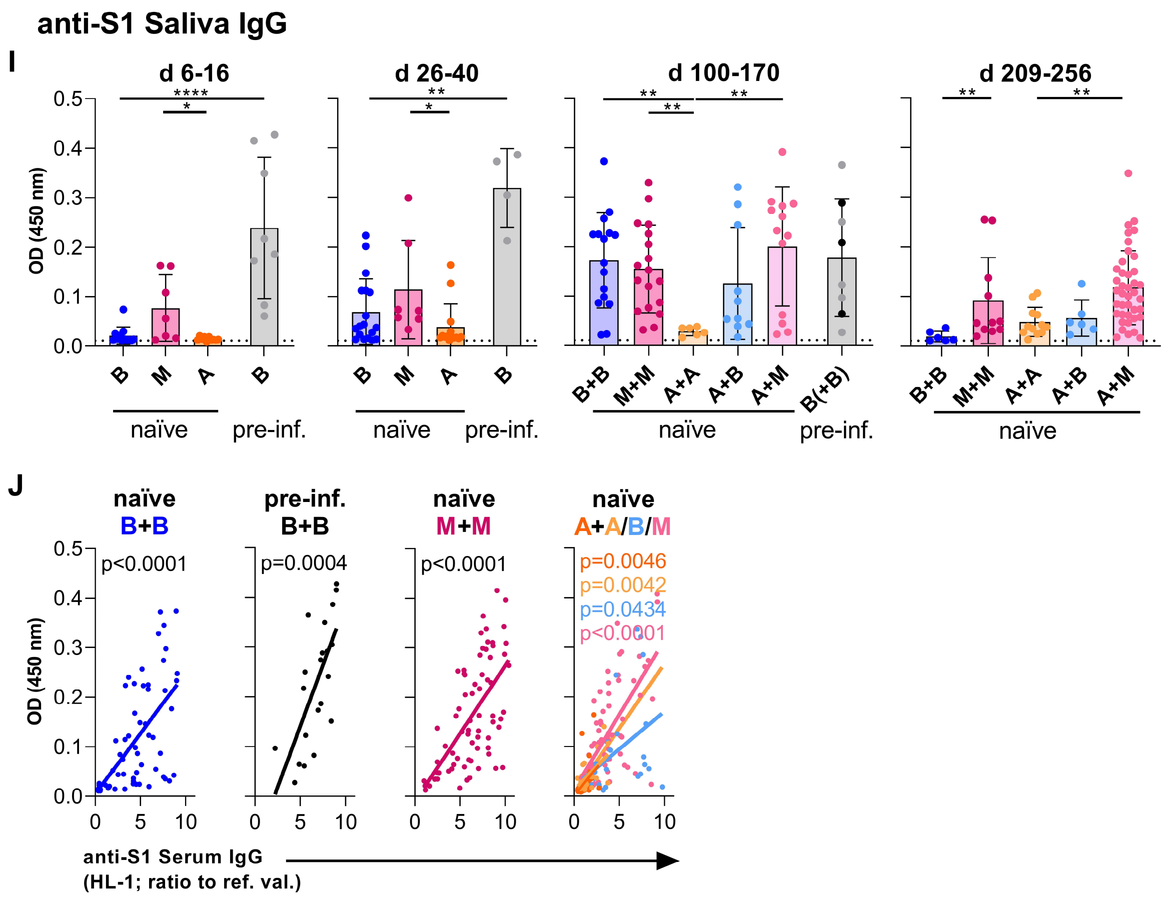

Fig. S1. Anti-S1 serum and saliva IgG levels.

(**A-C**) Anti-S1 serum IgG (EUROIMMUN (EI) ELISA) levels (ratio to reference values) of the indicated six vaccination groups. Gray bars: time windows of the second shot after the first shot with an mRNA (between day 21 and 45) or adenovirus-based (between day 70 and 84) vaccine. (**D**) Color legend of all six groups. (**E**) Anti-S1 serum IgG (EI ELISA) levels (the same y-axis as in (A)) of the six indicated groups for four different time intervals (data points from A-C). All samples of the second time interval (days 26-40) were taken before the second vaccination. Dashed (C) and dotted lines indicate the corresponding anti-S1 IgG average levels of pre-infected individuals without/ before vaccination or non-vaccinated healthy (negative) controls. (**F**) Correlations between anti-NCP serum IgG levels (ratio to reference value) and anti-S1 serum IgG (EI ELISA) levels (the same y-axis as in (A) and (E)) of the indicated groups. (**G**) Correlations between anti-S1 serum IgG (HL-1 ELISA) levels (data points from **Fig. 1A-C**) and anti-S1 serum IgG (EI ELISA) levels (the same y-axis as in (A), (E), and (F)) of the indicated groups. *p*-values of the indicated correlations are shown (the *p*-values for the naïve A+A/B/M groups are the same). (**H**) Anti-S1 serum IgG (HL-1 ELISA) levels of the indicated six groups for four different time intervals (data points from **Fig. 1A-C**). (**I**) Anti-S1 saliva IgG levels of the indicated six groups for four different time intervals (data

points from **Fig. 1E-G**). (**J**) Correlations between anti-S1 serum IgG (HL-1 ELISA) levels and anti-S1 saliva IgG levels. The same y-axis as in (I) and **Fig. 1E**. Statistics: Mann-Whitney test for the comparison of naïve and pre-infected (B(+B) vaccinees; Kruskal-Wallis test for the comparison of all naïve groups.

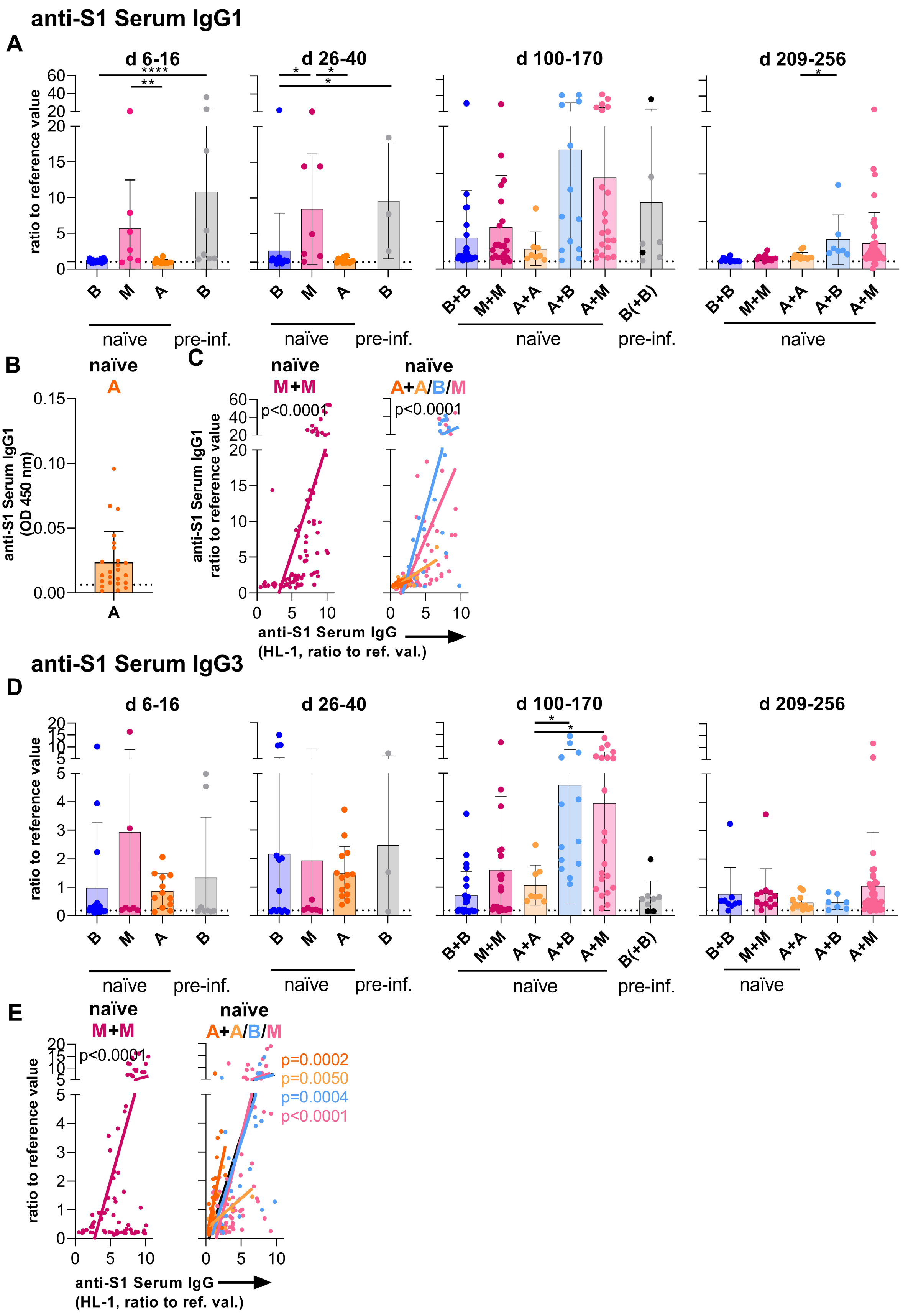

anti-S1 Serum IgG1
A
d 6-16
d 26-40
d 100-170
d 209-256
ratio to reference value
naïve
pre-inf.
B
naïve
A
anti-S1 Serum IgG1 (OD 450 nm)
C
naïve
M+M
p<0.0001
naïve
A+A/B/M
p<0.0001
anti-S1 Serum IgG1 ratio to reference value
anti-S1 Serum IgG
(HL-1, ratio to ref. val.)
anti-S1 Serum IgG3
D
d 6-16
d 26-40
d 100-170
d 209-256
ratio to reference value
naïve
pre-inf.
E
naïve
M+M
p<0.0001
naïve
A+A/B/M
p=0.0002
p=0.0050
p=0.0004
p<0.0001
ratio to reference value
anti-S1 Serum IgG
(HL-1, ratio to ref. val.)

Fig. S2. Anti-S1 serum IgG1 and IgG3 subclass levels.

(**A**) Anti-S1 serum IgG1 levels of the indicated six vaccination groups for four different time intervals (data points from **Fig. 2**). All samples of the second time interval (days 26-40) were taken before the second vaccination. (**B**) Anti-S1 serum IgG1 ELISA (OD 450 nm) data for individuals immunized one-time with AZD1222 (serum dilution 1/10). (**C**) Correlations between anti-S1 serum IgG (HL-1 ELISA) levels and anti-S1 serum IgG1 levels (extension to correlations in **Fig. 2**). *p*-values of the indicated correlations are shown (the *p*-values for naïve A+A/B/M groups are the same). (**D**) Anti-S1 serum IgG3 levels of the indicated six groups for four different time intervals (data points from **Fig. 2**). (**E**) Correlations between anti-S1 serum IgG (HL-1 ELISA) levels and anti-S1 serum IgG3 levels (extension to correlations in **Fig. 2**). Dotted lines indicate the corresponding anti-S1 IgG1 or 3 average levels of non-vaccinated healthy (negative) controls. Statistics: Mann-Whitney test for the comparison of naïve and pre-infected (B(+B) vaccinees; Kruskal-Wallis test for the comparison of all naïve groups.

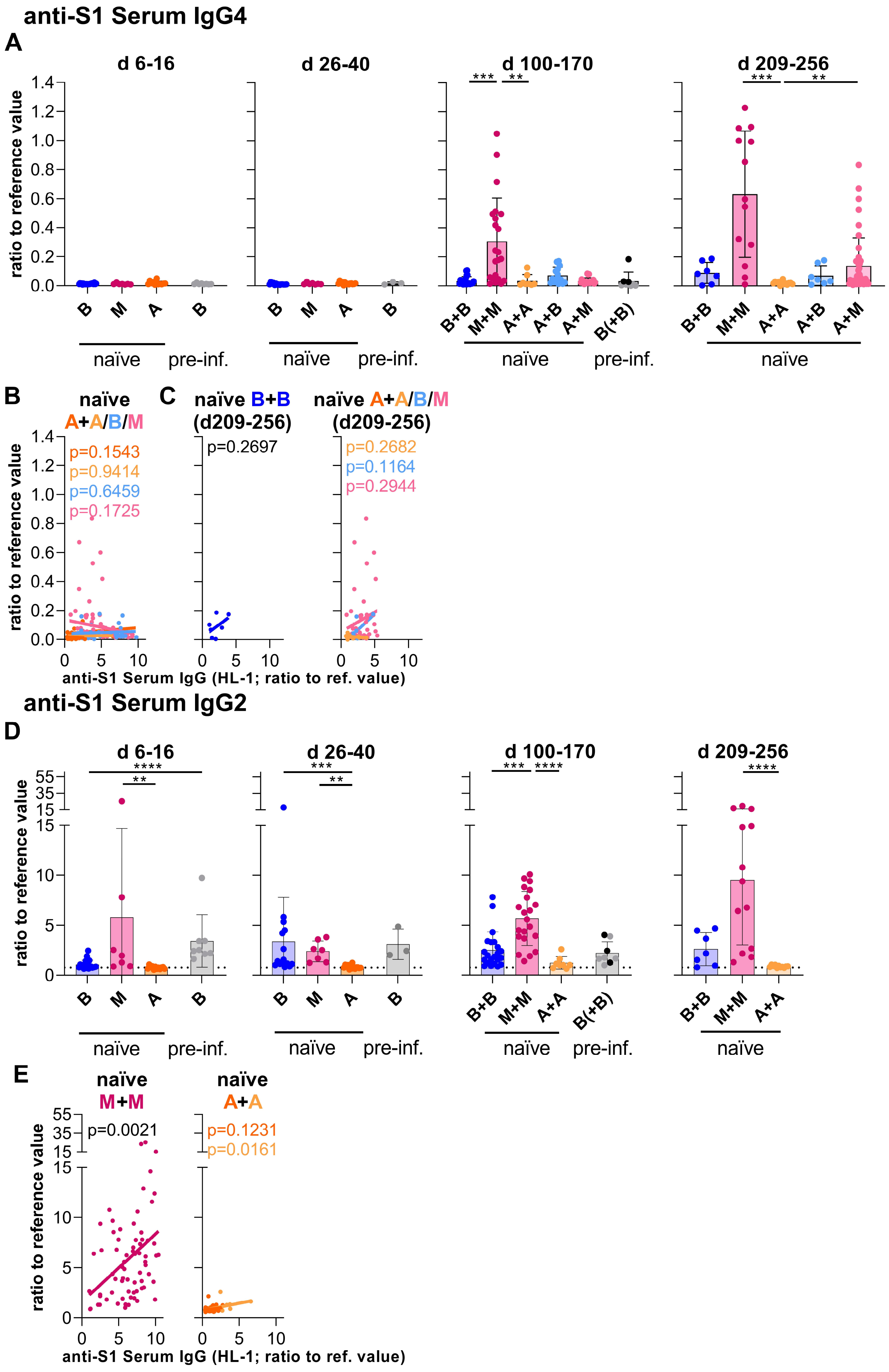
anti-S1 Serum IgG4
A
d 6-16
d 26-40
d 100-170
d 209-256
ratio to reference value
1.4
1.2
1.0
0.8
0.6
0.4
0.2
0.0
***
**
B
M
A
B+B
M+M
A+A
A+B
A+M
B(+B)
naïve
pre-inf.
B
naïve
A+A/B/M
p=0.1543
p=0.9414
p=0.6459
p=0.1725
C
naïve B+B
(d209-256)
p=0.2697
naïve A+A/B/M
(d209-256)
p=0.2682
p=0.1164
p=0.2944
0
5
10
anti-S1 Serum IgG (HL-1; ratio to ref. value)
anti-S1 Serum IgG2
D
55
35
15
10
5
****
E
naïve
M+M
p=0.0021
naïve
A+A
p=0.1231
p=0.0161

Fig. S3. Anti-S1 serum IgG4 and IgG2 subclass levels.

(**A**) Anti-S1 serum IgG4 levels of the indicated four groups for four different time intervals (data points from **Fig. 2**). All samples of the second time interval (days 26-40) were taken before the second vaccination. (**B**) Correlations between anti-S1 serum IgG (HL-1 ELISA) levels and anti-S1 serum IgG4 levels (extension to correlations in **Fig.2**). *p*-values of the indicated correlations are shown. (**C**) Correlations between anti-S1 serum IgG (HL-1 ELISA) levels and anti-S1 serum IgG4 levels between day (d) 209-256 (extension to correlations in **Fig. 2**). (**D**) Anti-S1 serum IgG2 levels of the indicated four groups for four different time intervals (data points from **Fig. 2**). (**E**) Correlations between anti-S1 serum IgG (HL-1 ELISA) levels and anti-S1 serum IgG2 levels (extension to correlations in **Fig. 2**). Dotted lines indicate the corresponding anti-S1 IgG2 or 4 average levels of non-vaccinated healthy (negative) controls. Statistics: Mann-Whitney test for the comparison of naïve and pre-infected B(+B) vaccinees; Kruskal-Wallis test for the comparison of all naïve groups.

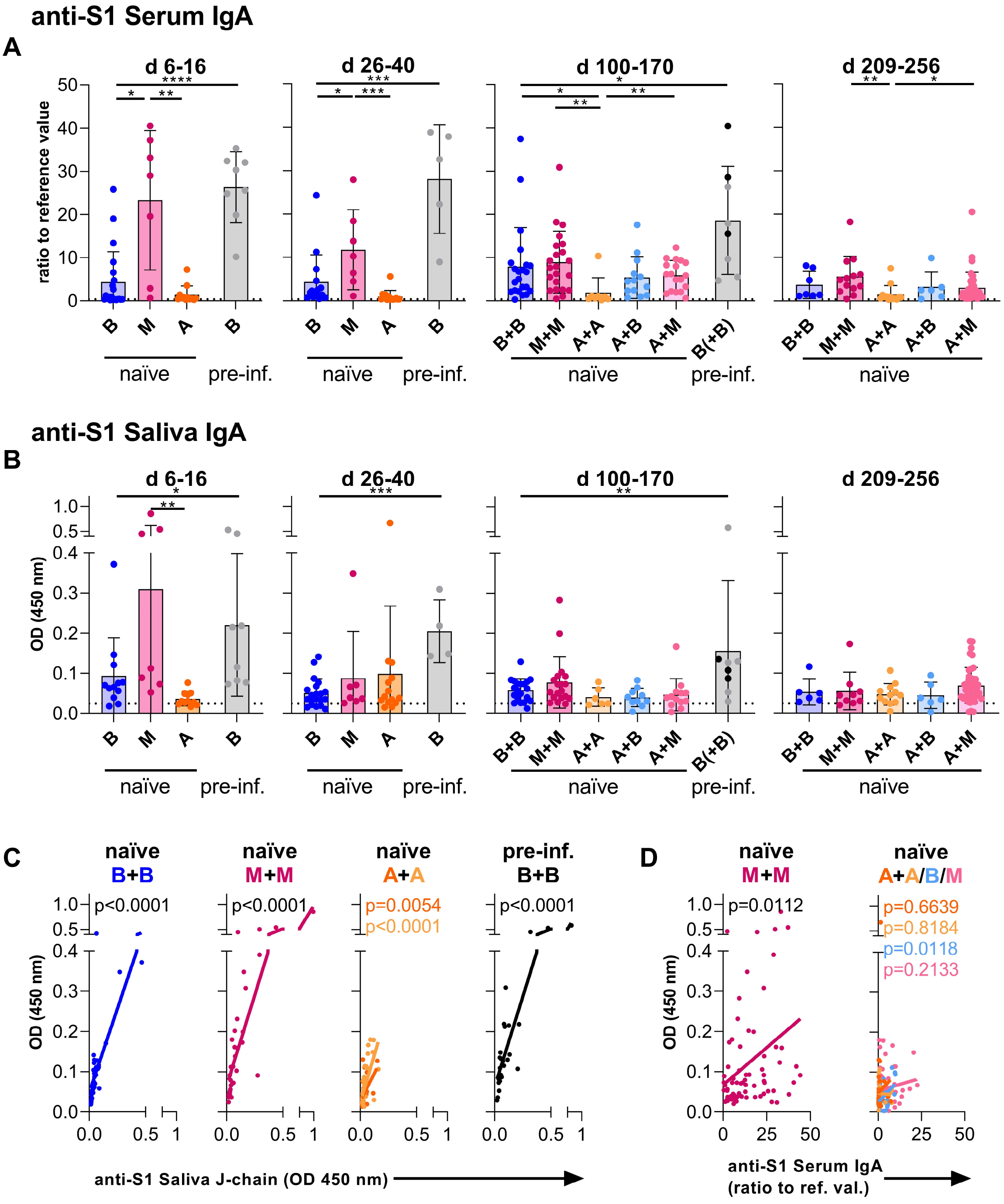

Fig. S4. Anti-S1 serum and saliva IgA levels.

(**A and B**) Anti-S1 (A) serum and (B) saliva IgA levels of the indicated six vaccination groups for four different time intervals (data points from **Fig. 3**). All samples of the second time interval (days 26-40) were taken before the second vaccination. Dotted lines indicate the corresponding anti-S1 serum or saliva IgA average levels of non-vaccinated healthy (negative) controls. (**C**) Correlations between anti-S1 saliva J-chain and anti-S1 saliva IgA levels. (**D**) Correlations between anti-S1 serum IgA and anti-S1 saliva IgA levels (extension to correlations

in **Fig. 3**) of the indicated groups. *p*-values of the indicated correlations are shown. Statistics: Mann-Whitney test for the comparison of naïve and pre-infected B(+B) vaccinees; Kruskal-Wallis test for the comparison of all naïve groups.

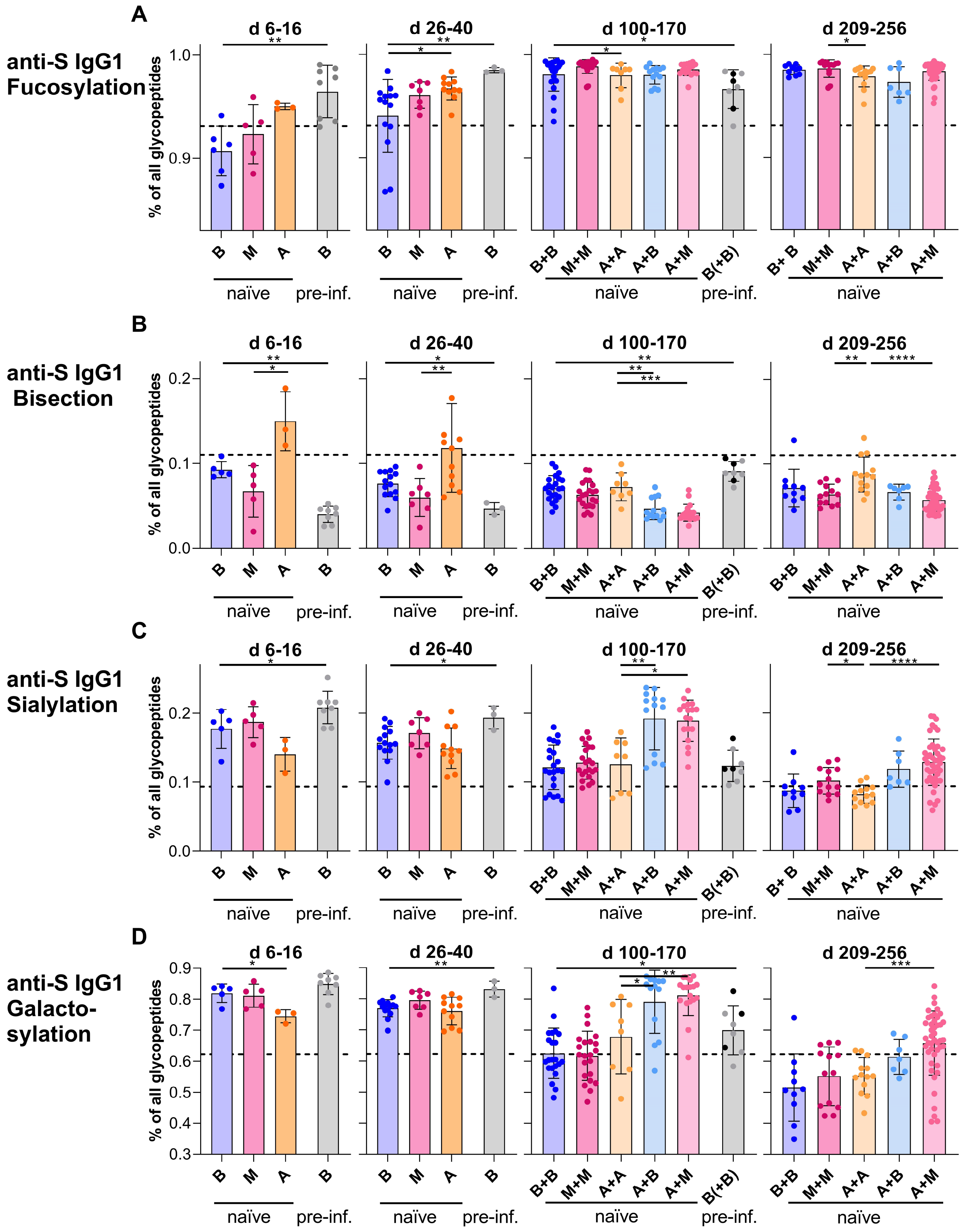
A
anti-S IgG1 Fucosylation
B
anti-S IgG1 Bisection
C
anti-S IgG1 Sialylation
D
anti-S IgG1 Galacto-sylation
d 6-16
d 26-40
d 100-170
d 209-256
% of all glycopeptides
B
M
A
B+B
M+M
A+A
A+B
A+M
B(+B)
naïve
pre-inf.

Fig. S5. Anti-S1 serum IgG1 Fc *N*-glycosylation

(**A-D**) Anti-S serum IgG1 Fc *N*- (A) fucosylation, (B) bisection, (C) sialylation, and (D) galactosylation of the indicated six vaccination groups for four different time intervals (data points from **Figs. 4 and 5**). All samples of the second time interval (days 26-40) were taken before the second vaccination. Dashed lines indicate the average level of total IgG1 Fc *N*-fucosylation, bisection, sialylation, or galactosylation, respectively (**Fig. S6**). Statistics: Mann-Whitney test for the comparison of naïve and pre-infected B(+B) vaccinees; Kruskal-Wallis test for the comparison of all naïve groups.

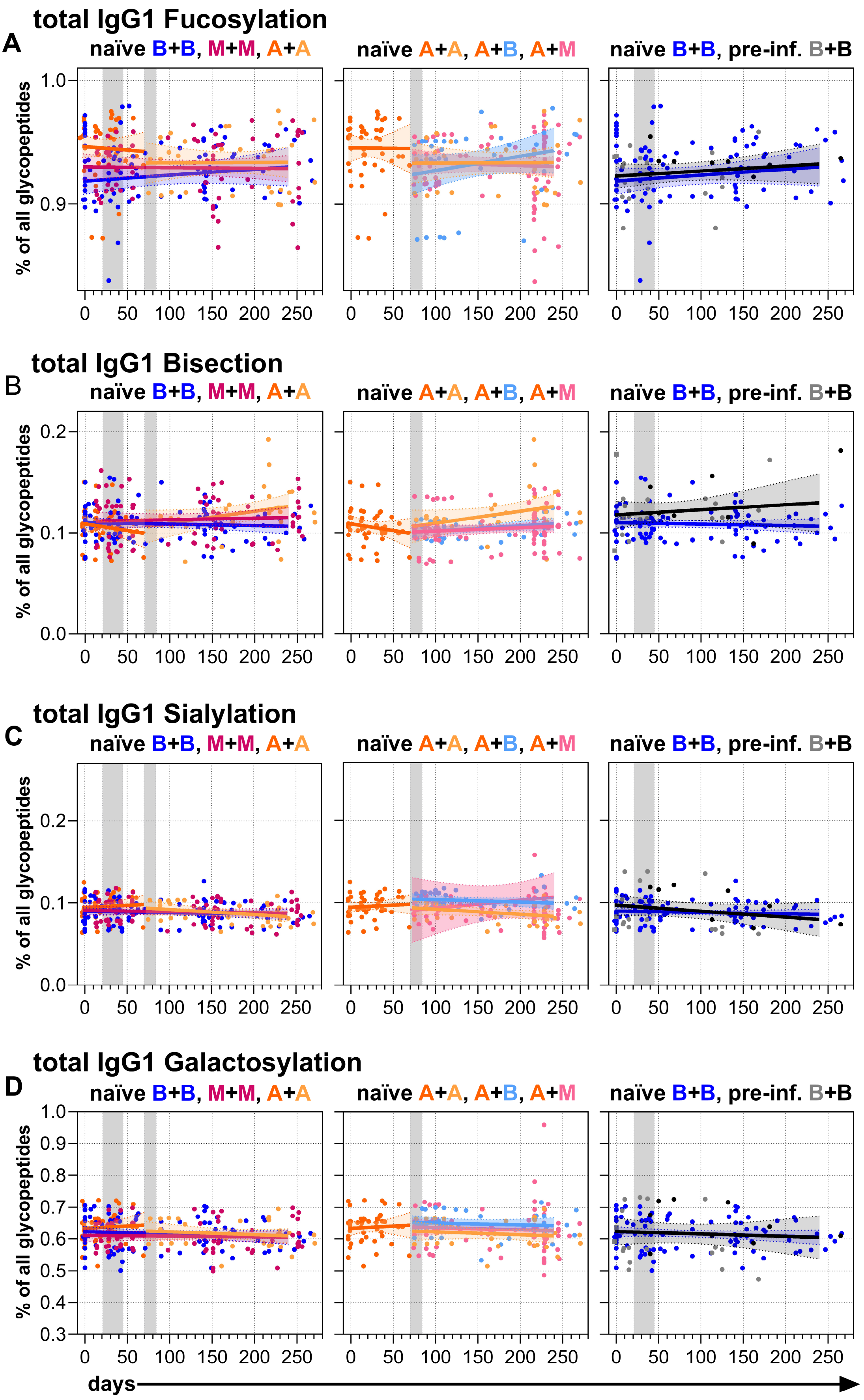

total IgG1 Fucosylation
A
naïve B+B, M+M, A+A
naïve A+A, A+B, A+M
naïve B+B, pre-inf. B+B
% of all glycopeptides
1.0
0.9
0 50 100 150 200 250
total IgG1 Bisection
B
0.2
0.1
0.0
total IgG1 Sialylation
C
total IgG1 Galactosylation
D
0.3
0.4
0.5
0.6
0.7
0.8
days

Fig. S6. Total serum IgG1 Fc *N*-glycosylation

(**A-D**) Total serum IgG1 Fc *N*- (A) fucosylation, (B) bisecting, (C) sialylation, and (D) galactosylation for the indicated six vaccination groups. The color legend is shown in **Fig. 1D**. Gray bars: time windows for the second shot after the first shot with an mRNA (between day 21 and 45) or adenovirus-based (between day 70 and 84) vaccine.

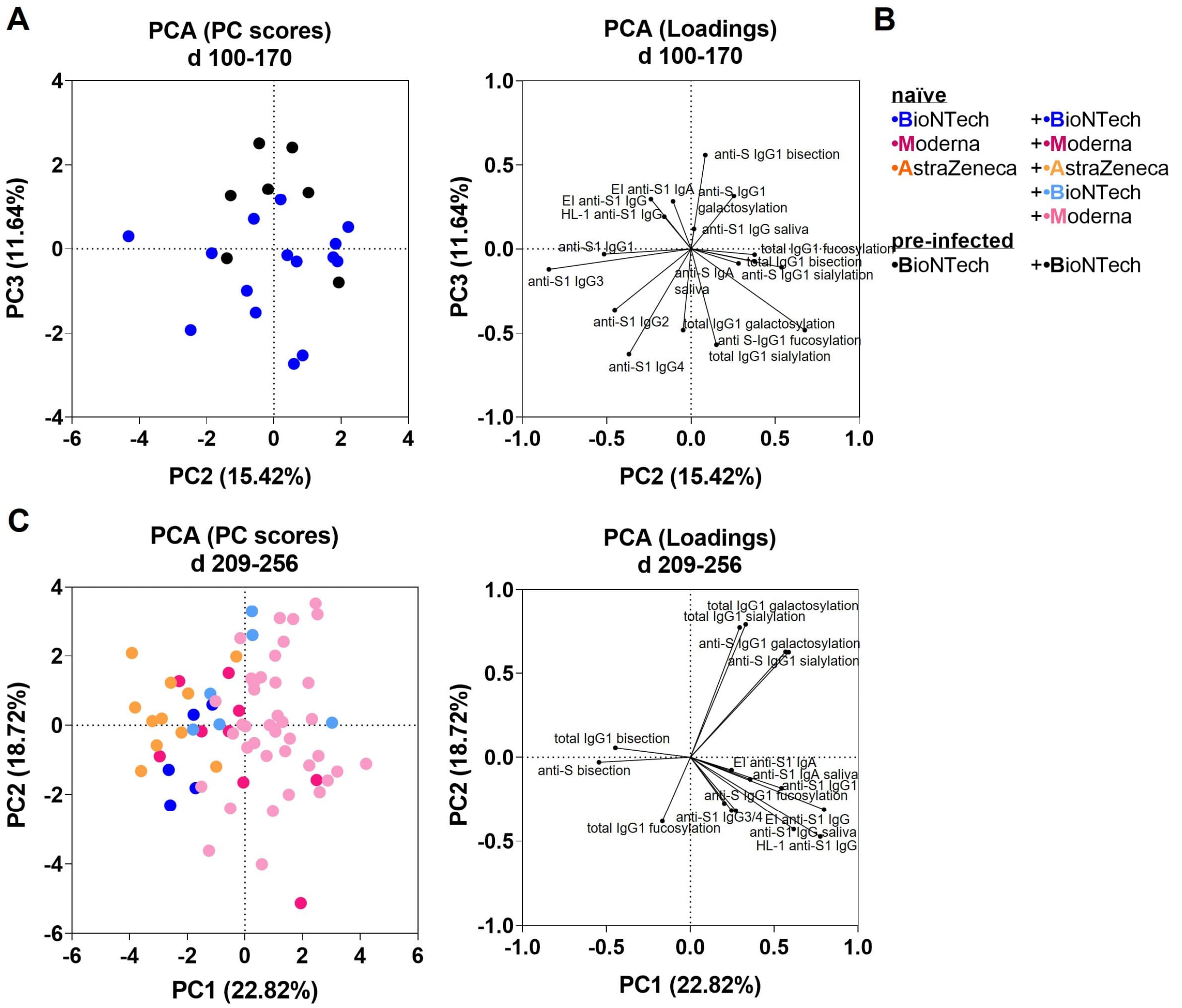

Fig. S7. Principal component analysis (PCA).

(A) PCA of data collected from naïve and pre-infected B(+B) vaccinated individuals between day (d) 100 and 170 upon first vaccination. Loadings as well as PC scores are shown. **(B)** Colour coding of all six vaccination groups. **(C)** PCA of data from all naïve vaccinated individuals between day 209 and 256 upon first vaccination.

Table S1: **Age- and sex-distributions of all vaccination groups.**

group→<br>variable↓	Naïve<br>BioNTech/Pfizer<br>+<br>BioNTech/Pfizer	Naïve<br>Moderna<br>+<br>Moderna	Naïve<br>AstraZeneca<br>+<br>AstraZeneca	Naïve<br>AstraZeneca<br>+<br>BioNTech	Naïve<br>AstraZeneca<br>+<br>Moderna	Pre-infected<br>BioNTech/Pfizer<br>+<br>BioNTech/Pfizer
**Age (years)**						
Average (Min-Max)	$\bar{X}$ 36<br>(19-66)	$\bar{X}$ 42<br>(22-61)	$\bar{X}$ 41<br>(22-62)	$\bar{X}$ 30<br>(23-44)	$\bar{X}$ 36<br>(22-63)	$\bar{X}$ 47<br>(27-74)
**Sex at Birth**						
Female	26	21	7	9	33	8
Male	22	4	7	3	11	6
Both	48	25	14	12	44	14

Table S2: IgG1 glycopeptides included in the final analyte list. Shown are the compositions, IgG glycan names according to alternative nomenclature, the monoisotopic mass of detected glycan species in the 2+ and 3+ charge, and proposed structures. The position and linkage of monosaccharides are proposed based on literature. In the glycan composition column, H: hexose; N: N-acetylhexosamine; F: fucose; S: N-acetylneuraminic acid (sialic acid).

**Glycan composition**	**Alternative nomen-clature**	**m/z $[M+2H]2^{+}$**	**m/z $[M+3H]3^{+}$**	**Proposed structure**	**Mean (of total IgG1 analysis)**	**SD (+/-)**	Compositions of hospitalized patients; raw glycopeptide peak data identified in the context of our previous study **(60)** that were used for calculation
**H3N4F1**	G0F	1317.527	878.687	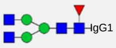	**16.32**	4.40	x
**H4N4**	G1	1325.524	884.018	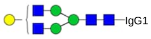	3.02	1.35	x
**H4N4F1**	G1F	1398.553	932.704	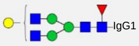	**30.36**	2.73	x
**H5N4**	G2	1406.550	938.036	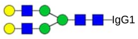	2.06	0.91	
**H5N4F1**	G2F	1479.579	986.722	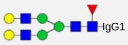	**21.08**	2.56	x
**H4N5F1**	G1FN	1500.093	1000.398	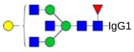	**8.24**	1.34	x
**H4N4F1S1**	G1FS1	1544.101	1029.736	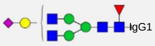	2.04	0.29	x
**H5N4S1**	G2S1	1552.098	1035.068	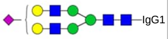	1.33	0.51	x
**H5N5F1**	G2FN	1581.119	1054.415	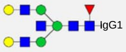	2.15	0.65	
**H5N4F1S1**	G2FS1	1625.127	1083.754	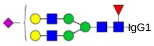	**12.54**	2.77	x
**H5N5F1S1**	G2FNS1	1726.668	1151.447	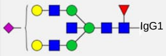	0.43	0.13	x
**H5N4F1S2**	G2FS2	1770.675	1180.786	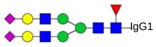	0.43	0.13	x
**sum**					100%		100%

Table S3: Description and calculation of IgG1 glycosylation traits.

Derived trait	Description	Formula
**fucosylation**	*N*-glycans carrying a core fucose	(H3N4F1 + H4N4F1 + H5N4F1 + H4N5F1 + H5N5F1 + H4N4F1S1 + H5N4F1S1 + H5N4F1S2 + H5N5F1S1) / sum of all glycopeptides
**bisection**	*N*-glycans carrying a bisected N-acetylglucosamine	(H4N5F1 + H5N5F1 + H5N5F1S1) / sum of all glycopeptides
**galactosylation**	*N*-glycans carrying a galactose	(1/2 * (H4N4 + H4N4F1 + H4N5F1 + H4N4F1S1) + 2/2 * (H5N4 + H5N4F1 + H5N5F1 + H5N4S1 + H5N4F1S1 + H5N4F1S2 + H5N5F1S1)) / sum of all glycopeptides
**sialylation**	*N*-glycans carrying an N-acetylneuraminic acid (sialic acid)	(1/2 * (H5N4S1 + H4N4F1S1 + H5N4F1S1 + H5N5F1S1) + 2/2 * H5N4F1S2) / sum of all glycopeptides
